# Rethinking Domain‐Specific Pretraining by Supervised or Self‐Supervised Learning for Chest Radiograph Classification: A Comparative Study Against ImageNet Counterparts in Cold‐Start Active Learning

**DOI:** 10.1002/hcs2.70009

**Published:** 2025-04-06

**Authors:** Han Yuan, Mingcheng Zhu, Rui Yang, Han Liu, Irene Li, Chuan Hong

**Affiliations:** ^1^ Duke‐NUS Medical School, Centre for Quantitative Medicine Singapore Singapore; ^2^ Department of Engineering Science University of Oxford Oxford UK; ^3^ Department of Computer Science Vanderbilt University Nashville Tennessee USA; ^4^ Information Technology Center University of Tokyo Bunkyo‐ku Japan; ^5^ Department of Biostatistics and Bioinformatics Duke University Durham North Carolina USA

**Keywords:** chest radiograph analysis, cold‐start active learning, COVID‐19, psychiatric pneumonia, radiology foundation model

## Abstract

**Objective:**

Deep learning (DL) has become the prevailing method in chest radiograph analysis, yet its performance heavily depends on large quantities of annotated images. To mitigate the cost, cold‐start active learning (AL), comprising an initialization followed by subsequent learning, selects a small subset of informative data points for labeling. Recent advancements in pretrained models by supervised or self‐supervised learning tailored to chest radiograph have shown broad applicability to diverse downstream tasks. However, their potential in cold‐start AL remains unexplored.

**Methods:**

To validate the efficacy of domain‐specific pretraining, we compared two foundation models: supervised TXRV and self‐supervised REMEDIS with their general domain counterparts pretrained on ImageNet. Model performance was evaluated at both initialization and subsequent learning stages on two diagnostic tasks: psychiatric pneumonia and COVID‐19. For initialization, we assessed their integration with three strategies: diversity, uncertainty, and hybrid sampling. For subsequent learning, we focused on uncertainty sampling powered by different pretrained models. We also conducted statistical tests to compare the foundation models with ImageNet counterparts, investigate the relationship between initialization and subsequent learning, examine the performance of one‐shot initialization against the full AL process, and investigate the influence of class balance in initialization samples on initialization and subsequent learning.

**Results:**

First, domain‐specific foundation models failed to outperform ImageNet counterparts in six out of eight experiments on informative sample selection. Both domain‐specific and general pretrained models were unable to generate representations that could substitute for the original images as model inputs in seven of the eight scenarios. However, pretrained model‐based initialization surpassed random sampling, the default approach in cold‐start AL. Second, initialization performance was positively correlated with subsequent learning performance, highlighting the importance of initialization strategies. Third, one‐shot initialization performed comparably to the full AL process, demonstrating the potential of reducing experts' repeated waiting during AL iterations. Last, a U‐shaped correlation was observed between the class balance of initialization samples and model performance, suggesting that the class balance is more strongly associated with performance at middle budget levels than at low or high budgets.

**Conclusions:**

In this study, we highlighted the limitations of medical pretraining compared to general pretraining in the context of cold‐start AL. We also identified promising outcomes related to cold‐start AL, including initialization based on pretrained models, the positive influence of initialization on subsequent learning, the potential for one‐shot initialization, and the influence of class balance on middle‐budget AL. Researchers are encouraged to improve medical pretraining for versatile DL foundations and explore novel AL methods.

## Graphical abstract

This paper contributes a systematic analysis to examine the effectiveness of domain‐specific models against their ImageNet counterparts in cold‐start AL, proposes a representation‐based uncertainty sampling for AL initialization, and identifies key findings related to AL stages, backbone architectures, and AL paradigms.

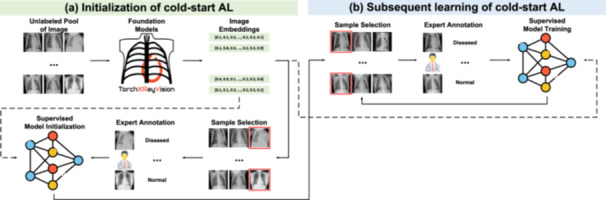

**Abbreviations**
AL: active learning
AUPRC: area under the precision‐recall curve
AUROC: area under the receiver operating characteristic curve
DL: deep learning
MC: Monte Carlo
MLP‐3: three‐layer multilayer perceptron
REMEDIS: Robust and Efficient MEDical Imaging with Self‐supervision
SGD: stochastic gradient descent
TXRV: TorchXRayVision

## 1 Background

This section begins by motivating the use of active learning (AL) to reduce annotation costs in deep learning (DL) models, especially its orthogonal value compared to other strategies for DL on limited annotated samples, including data augmentation, transfer learning, and semisupervised learning. We then differentiate between warm‐start and cold‐start AL, highlighting that cold‐start AL better addresses real‐world scenarios. Next, we illustrate common strategies and related work for cold‐start AL, focusing on both the initialization and subsequent learning stages. After that, we demonstrate the potential of domain‐specific pretrained models, also known as foundation models, in enhancing cold‐start AL. Finally, we summarize our key contributions.

### 1.1 Motivation

DL has achieved remarkable success in chest radiograph analysis [1, 2, 3], but its performance heavily relies on large volumes of chest radiographs and high‐quality diagnostic annotations [4, 5]. Unlike natural scene labeling, which primarily relies on common sense [6, 7] and can leverage crowdsourcing platforms [8], chest radiograph annotation requires specialized expertise [9], making it time‐consuming and cost‐intensive [10]. To reduce annotation costs, alleviate clinician workload, and optimize computational resources by avoiding redundant data [11, 12, 13], AL has been proposed to iteratively select a small subset of data points whose annotations are most beneficial for model convergence, querying these labels from experienced medical professionals as oracles [10, 14, 15].

Although various strategies have been proposed to address DL under the constraint of limited annotated samples, AL offers a distinct and irreplaceable advantage worthy of focused investigation. A commonly considered approach is data augmentation, which involves applying transformations to existing labeled data. However, this strategy potentially fails to introduce truly novel or representative information and can even degrade DL performance when augmented samples are physically implausible or semantically meaningless [16]. In contrast, AL selects genuine data samples, avoiding the reinforcement of existing biases, and the misrepresentation of real‐world properties. Another popular technique is transfer learning, which leverages a model pretrained on a source data set for the target data set. However, transfer learning can suffer from distribution mismatches between the source and target data sets [17]. Even with fine‐tuning on small annotated samples, biases inherent in the source data set are often challenging to mitigate [18]. Under the same annotation budget, AL directly optimizes the model for the target data set, ensuring more efficient use of resources. A third approach is semisupervised learning, which builds on limited annotated data by exploiting the structure of unlabeled data. However, if the initial labeled data set is poorly representative, semisupervised learning risks propagating errors and failing to generalize effectively [19]. AL, however, dynamically adapts to the model's learning state and iteratively refines the model through active querying, thus mitigating the risks of error propagation and poor generalization. It is important to highlight that this comparison aims to underscore AL's complementary value rather than diminish the utility of other methods. Indeed, these techniques can be integrated with AL to enhance performance, as suggested in prior studies [20, 21, 22]. In this work, we focus exclusively on AL to systematically investigate the potential of AL in the context of recent advancements in foundation models.

### 1.2 Related Work

#### 1.2.1 Warm‐Start and Cold‐Start AL

AL methods can be broadly classified into warm‐start and cold‐start AL, depending on the initialization stage [23]. Specifically, warm‐start AL typically involves two stages: an initial phase where the model is trained on a small, preselected, annotated subset of images, and a subsequent learning phase where various query strategies are employed to select additional images for annotation and model fine‐tuning based on the trained model [24]. Cold‐start AL also comprises two stages, but unlike warm‐start AL, it begins without any annotated samples. Instead, it autonomously selects initial samples, sends them to oracles for annotation, and proceeds with model training [25].

Although warm‐start AL is commonly studied and has been applied to a spectrum of clinical tasks such as breast mass localization [9], white matter tract segmentation [10], optical coherence tomography segmentation [26], and so on [6, 7, 27, 28, 29, 30, 31, 32, 33], it requires preselection of sample annotation belonging to diverse classes in the initial stage. This reliance is often impractical in real‐world AL scenarios, where none of the samples in a new data set are labeled, making it impossible to prepare representative instances for each category [34], especially in medical scenarios with a class‐skewed distribution [35]. Therefore, cold‐start AL is more suited to real‐world applications and becomes the focus of our study.

#### 1.2.2 Cold‐Start AL Strategies

The primary challenge in cold‐start AL lies in the initialization phase: how to select annotation‐worthy samples that cover diverse classes and significantly contribute to model convergence in the absence of label information. In other words, how can raw image pixels be utilized to identify samples that merit labeling? Upon completion of the initial sample selection and annotation, standard warm‐start strategies can be employed because the model, following the initialization stage, has developed sufficient competency on the target data and task, thereby satisfying the prerequisites for warm‐start AL.

For the initialization stage, random sampling is often the first method considered by researchers. Although this method works well on balanced data sets, it typically requires selecting a large number of instances to capture all potential classes in imbalanced scenarios [35], which is impractical for AL formulations and overlooks the informative sample features [36]. To address these limitations, diversity sampling, also known as representativeness sampling, has been proposed. This method selects samples that are representative of the underlying data distribution of diverse classes [31] based on the modeling of raw image pixels [37] in an unsupervised or self‐supervised manner. For example, He et al. proposed a two‐stage clustering approach to address the cold‐start problem in AL initialization, which is adaptable to class imbalance [35]. In the first stage, the density peak clustering algorithm [38] was used to separate samples from majority and minority classes into distinct clusters. In the second stage, a cluster‐adaptive method was employed to identify the most representative samples within each cluster. This approach effectively selects samples that improve both class coverage and model performance.

For the subsequent learning stage, previous initialization methods, such as random sampling [39] and diversity [40], can also be employed. These approaches do not rely on annotation information or models developed from the initialization phase, whereas other methods for subsequent learning typically do. Due to its simplicity [41] and outperformance [42], uncertainty sampling, also known as informativeness sampling, is the most widely used approach [31]. Specifically, this method selects data points where the current model exhibits the greatest uncertainty, often those near the decision boundary [41]. Common uncertainty metrics include margin of confidence, least confidence, and entropy [43, 44]. Uncertainty sampling can be further integrated with diversity or other strategies to form hybrid methods [23, 45, 46, 47]. For instance, Yang et al. proposed an annotation suggestion method that integrates uncertainty and diversity [48]. They first calculated the variance across a set of bootstrap‐aggregated models [49], and then identified high‐variance unlabeled samples [50]. Among these, the samples with the highest similarity sum to all other unlabeled samples were deemed representative and selected for annotation. Shen et al. introduced a three‐step integrative strategy to gradually identify the most informative samples [51]. First, they selected a large subset with the highest uncertainty based on Monte Carlo (MC) dropout [52]. Next, they refined the subset by retaining samples that could represent the entire unlabeled set. Finally, they excluded samples already similar to annotated data. An alternative to multistep integration is the use of weighted combinations of different metrics in a single step [53]. For example, Mahapatra et al. computed sample informativeness as a weighted sum of entropy‐based uncertainty and the mean squared distance between the feature vectors of candidate images and all other unlabeled samples [54].

#### 1.2.3 Domain‐Specific Pretraining for Cold‐Start AL

In chest radiograph analysis, pretraining plays a crucial role in reducing the need for large training data sets while improving model performance. Traditionally, pretraining involves the collection and annotation of large‐scale data sets similar to the target data set, followed by supervised learning to develop DL models with optimal initial parameters for downstream tasks. However, the rapid growth of unlabeled data has outpaced the capacity of experts to provide annotations. To address this, researchers have introduced self‐supervised learning, which exploits the inherent structure and relationships within the data to derive effective initial parameters. Self‐supervised learning has been deployed on large‐scale medical data sets that span different levels of specificity, from organ‐ or task‐specific models such as those for abdominal organs [55] or sight‐threatening eye diseases [56], to domain‐specific models like those for chest radiographs [57, 58], and even general models capable of handling multiple domains, including dermatology photographs, fundus imaging, digital pathology, chest radiographs, and mammography [59]. Both supervised and self‐supervised models can generate low‐dimensional yet information‐rich representation vectors for external data sets from the same target domains that they were not trained on. These numeric representations provide one of the overarching advantages of pretrained models, serving as feature inputs for downstream specialized models. Therefore, we refer to these domain‐specific pretrained models, whether derived from supervised or self‐supervised learning, as foundation models, and use this term interchangeably with domain‐specific pretrained models in the following sections. By reducing the dimensionality relative to the original images, foundation models allow for more compact model parameters and lower the computational cost of model training [60].

Foundation model‐based representations hold significant potential for use in the initialization and subsequent learning stages of cold‐start AL. In the initialization phase, clustering is a common diversity sampling method, but it often faces convergence challenges due to the high dimensionality of original image pixel features [61]. These challenges can be mitigated by employing low‐dimensional representation vectors [62]. Additionally, these representations can replace raw image pixels in model design, enabling more efficient parameterization during both the initialization and subsequent training stages. For instance, researchers applied the BERT foundation model [63] to address cold‐start sentence classification [64]. They encoded samples into novel vectors that captured diversity through hidden representations and uncertainty via model confidence scores. Based on these vectors, they used K‐means++ clustering [65] to select initial samples for annotation.

However, pretraining is not a novel concept in the DL domain. Before the advent of domain‐specific foundation models, various DL models pretrained on ImageNet [66], a general domain data set with human annotation, had already been applied to diverse external data sets [67, 68], demonstrating the ability to generate informative representations [69]. As such, ImageNet pretrained backbones should be considered valuable counterparts to foundation models, particularly because many foundation models, such as CXR foundation [57], leverage classic network architectures like ResNet [70], which also offer ImageNet pretrained versions. Therefore, a thorough evaluation of foundation models and their influence on cold‐start AL is essential to understanding the capability of these models, inspiring both new application scenarios for foundation models and the development of novel AL methods in the era of self‐supervised learning.

### 1.3 Contributions

In this work, we provide the following contributions. First, we contribute a systematic, quantitative, and reproducible analysis to examine the effectiveness of domain‐specific pretrained models against their ImageNet counterparts in both the initialization and subsequent learning stages of cold‐start AL. Second, we propose a representation‐based uncertainty sampling in the initialization stage of cold‐start AL to address the difficulty that uncertainty sampling strategies have no access to sample labels in the initialization stage. Third, we conduct rigorous statistical tests to reveal the relationship between AL initialization and subsequent learning, the comparison between the lightweight representation‐based model and raw image‐based model, and the comparability of one‐shot initialization and the complete AL under the same annotation budget. Last, we implement correlation tests to identify the impact of class balance of initialization samples on AL initialization and subsequent learning.

## 2 Methods

In this section, we begin by introducing key notations and presenting a general formulation for cold‐start AL, encompassing both the initialization and subsequent learning stages. We then detail specific strategies for each stage, leveraging domain‐specific foundation models and their ImageNet counterparts. We focus primarily on binary image classification to align with the real‐world experiments in the following section.

### 2.1 Cold‐Start AL Formulation

#### 2.1.1 Initialization

We denote the initial unlabeled and labeled image data sets as $D_{0}^{u}$ and $D_{0}^{l}$, respectively. Before the initialization, $D_{0}^{l}=\emptyset$ is an empty set, and the unlabeled pool $D_{0}^{u}$ contains $N_{0}^{u}$ unlabeled two‐dimensional images $I_{i}$ with the width of $W_{0}$ and the height of $H_{0}$. Then a query strategy $Q_{0}$ is leveraged to select $N_{0}^{l}$ images $I_{j}$ to be annotated by oracles based on original image pixels. After that, $D_{0}^{u}$ is updated into $D_{0}^{u}\langle (I_{j}),j=1,2,\ldots ,N_{0}^{l}\rangle$ by removing the selected images $\langle {(I}_{j}),j=1,2,\ldots ,N_{0}^{l}\rangle$ and professional medical experts such as radiologists give binary labels $Y_{j}$ to each of the selected images $I_{j}$, updating $D_{0}^{l}$ into $\langle \left(I_{j},Y_{j}\right),j=1,2,\ldots ,N_{0}^{l}\rangle$. Based on the $D_{0}^{l}$ consisting of samples and corresponding annotations, a classifier $M_{c}$ is trained to learn the projection from $I_{j}$ to $Y_{j}$. When the training of $M_{c}$ is completed, the initialization stage of cold‐start AL is finished and proceeded into the next stage of subsequent learning.

#### 2.1.2 Subsequent Learning

Different from the initialization stage without any annotation information, subsequent learning has a classifier $M_{c}$ with certain discriminability on the target task and therefore can use a classifier‐based informative sampling strategy $Q_{1}$. Assuming a total annotation budget $B=N_{0}^{l}+k\times N_{1}^{l}$, in each query iteration τ=1,2,3,…,k, the subsequent learning strategy $Q_{1}$ select $N_{1}^{l}$ samples $I_{s}$ from the unlabeled pool $D_{\tau -1}^{u}$ and send them to oracles for annotation, forming an annotated set $D_{\tau }^{l}=D_{\tau -1}^{l}\cup \langle \left(I_{s},Y_{s}\right),s=1,2,\ldots ,N_{1}^{l}\rangle$. Meanwhile, the unlabeled pool $D_{\tau }^{u}=D_{\tau -1}^{u}\backslash \langle (I_{s}),s=1,2,\ldots ,N_{1}^{l}\rangle$is updated by removing the selected images $I_{s}$. Based on the updated annotated set $D_{\tau }^{l}$, the classifier $M_{c}$ can be further trained, and upon completion, the subsequent learning process can advance to the next iteration.

### 2.2 Initialization Strategy

**Figure 1:**
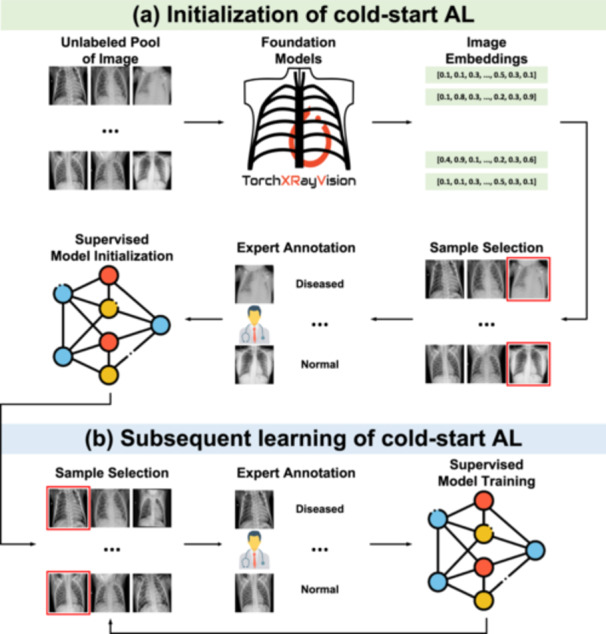
Cold‐start AL workflow based on domain‐specific foundation models. (a) The initialization stage. (b) The subsequent learning stage. After sample selection and expert annotation, supervised model training can be conducted using either the original image pixels or embeddings derived from foundation models as classifier inputs.

In the previous subsection, we introduced a general formulation for cold‐start AL. Here, we present three different strategies based on foundation models: diversity sampling, uncertainty sampling, and hybrid sampling. Additionally, we discuss random sampling, a common approach that does not require a pretrained model. Figure 1a illustrates the cold‐start AL initialization process for binary disease diagnoses. Unlike the general formulation, the three strategies require a foundation model $M_{f}$ to process images $I_{i}$ into embeddings $E_{i}$ which has much lower dimensions than the image dimension of $W_{0}\times H_{0}$. Also, previous literature [57, 71] has demonstrated that $E_{i}$ can replace $I_{i}$ as model inputs, enabling the development of a simplified model $M_{c}^{\prime }$ with comparable or superior performance, as depicted by the dashed line in Figure 1a.

#### 2.2.1 Diversity Sampling

The core of AL is to select informative samples, though the precise definition of informativeness remains an open question [9]. Some researchers suggest that an effective strategy, known as diversity sampling, is to select images that are representative of the overall data set while avoiding redundancy from visually similar images [51]. Among various diversity sampling strategies, clustering methods are considered classic approaches [72, 73, 74, 75]. These methods have been validated as effective for partitioning chest radiograph data sets into distinct clusters based on image features [76]. The centroids of each cluster are deemed diverse, as they originate from different clusters, and representative, as they serve as the central points of these clusters [77, 78]. We select K‐means [79] as the clustering method and follow previous studies that split the same amount of clusters as the annotation budget [27, 80, 81, 82]. Formally, the query strategy $Q_{0}$ divides $D_{0}^{u}=\langle {(I}_{i}),i=1,2,\ldots ,N_{0}^{u}\rangle$ into $N_{0}^{l}$ subgroups through $E_{i}^{u}$‐based K‐means, selects the centroid sample $I_{j}$ from each cluster, and sends the selected samples to oracles for annotation to constitute $D_{0}^{l}=\langle \left(I_{j},Y_{j}\right),j=1,2,\ldots ,N_{0}^{l}\rangle$.

#### 2.2.2 Uncertainty Sampling

In contrast to diversity sampling, which aims to select representative samples, an alternative approach, called uncertainty sampling, emphasizes the selection of the most uncertain samples for the current model, positing that these samples contribute most to model convergence. In the initialization stage, sample uncertainty for the target task is unavailable. Nath et al. [83] introduced a proxy task for image segmentation using morphological operations and employed MC dropout to estimate sample uncertainty [52]. However, their approach was restricted to computed tomography. In contrast, we developed a more generalizable auxiliary task based on foundation models to enable the computation of sample uncertainty across a broader range of applications.

Given that domain‐specific pretrained representation $E_{i}$ encapsulates high‐density information from the original image $I_{i}$ [84, 85, 86], enabling a range of downstream tasks [87, 88, 89], we hypothesize that $E_{i}^{u}$ can serve as an auxiliary prediction target. If a model exhibits uncertainty in predicting $E_{i}$, this suggests that the mapping between $I_{i}$ and the lower dimensional $E_{i}$ is challenging. Consequently, such samples are likely to be difficult for downstream tasks, including the target task. Formally, based on the image‐representation pair $\langle {(I}_{i},E_{i}),i=1,2,\ldots ,N_{0}^{u}\rangle$, we adopt the same architecture of $M_{c}$ and modify its final layer to match the dimension of $E_{i}$, generating the auxiliary model $M_{u}$ for uncertainty estimation. When the training of $M_{u}$ is completed, we follow the previous studies [51, 90, 91, 92, 93] and use MC dropout to approximate sample uncertainty [52]. Specifically, the trained model $M_{u}$ takes $I_{i}$ as the input and feedforward it T times. In iteration φ, a random dropout pattern is activated with a probability of P, and the model output $M_{u,\varphi }(I_{i})$ is recorded. Based on the aggregation set $\langle (M_{u,\varphi }(I_{i})),\varphi =1,2,\ldots ,T\rangle$, the inference variance is calculated via $(1/T)\sum_{\varphi =1}^{T}{\left(M_{u,\varphi }\left(I_{i}\right)-(1/T)\sum_{\varphi =1}^{T}M_{u,\varphi }\left(I_{i}\right)\right)}^{2}$. A large variance demonstrates that the process of $I_{i}$ by $M_{u}$ is either highly sensitive to neuron connectivity altering or akin to random guessing, reflecting significant uncertainty [51, 93]. The $I_{i}$ with the highest uncertainty, that is, inference variance, are selected and sent to oracles for annotation to constitute $D_{0}^{l}=\langle \left(I_{j},Y_{j}\right),j=1,2,\ldots ,N_{0}^{l}\rangle$.

#### 2.2.3 Hybrid Sampling of Diversity and Uncertainty

Diversity sampling selects representative samples while within a limited budget of AL, it might choose uninformative samples that are easy to distinguish and contribute marginally to model capability [94]. Uncertainty sampling suffers from selecting redundant samples to be labeled due to similar high uncertainty values [95] and a potential improvement could be a hybrid method to identify highly diverse and uncertain samples to convey more information with the same amount of annotated data [96, 97].

We employ a classic two‐step hybrid method [81, 98, 99] to first partition $D_{0}^{u}=\langle {(I}_{i}),i=1,2,\ldots ,N_{0}^{u}\rangle$ into $N_{0}^{l}$ subgroups through $E_{i}^{u}$‐based K‐means and then from each cluster, selects the most uncertain $I_{i}$ with the highest inference variance calculated by MC dropout as detailed in the previous subsection. This two‐step approach ensures the selection of diverse and uncertain samples to construct $D_{0}^{l}$ comprising $N_{0}^{l}$ samples along with their annotations.

#### 2.2.4 Random Sampling

In addition to the three initialization strategies based on domain‐specific foundation models, random sampling remains the most widely used and traditional method, as shown in previous studies [6, 7, 10, 27, 28, 29, 30, 31, 32, 33, 74]. Although commonly employed, random sampling is not without limitations. For instance, prior research has demonstrated that it does not ensure the informativeness of the initial samples selected for annotation, which may negatively impact downstream AL performance [10]. Moreover, random sampling is prone to issues related to data imbalance, especially during initialization, where selecting minority samples can require a substantial budget [35, 100].

### 2.3 Subsequent Learning Strategy

Upon completion of the initial sample selection and annotation, a model with sufficient competency on the target data and task becomes available, and based on the model, we can step into the subsequent learning stage of cold‐start AL, as depicted in Figure 1b. Following the initial sample selection and annotation, a discriminative model $M_{c}$ or $M_{c}^{\prime }$ is developed, enabling the next stage of subsequent learning. Although a range of warm‐start strategies could be applied at this stage, our study focuses on comparing foundation models with their ImageNet counterparts. We therefore employ classic uncertainty sampling strategies [100], which have demonstrated strong performance in prior studies [78, 101, 102, 103]. It is important to note that the uncertainty in this phase, given the availability of a discriminative model, differs from that discussed during the initialization stage, which will be further elaborated in the following paragraph.

In the context of subsequent learning, uncertainty sampling encompasses three primary methods: least confidence sampling, margin of confidence sampling, and entropy‐based sampling [30]. Notably, these methods converge on the same conclusion: the most uncertain samples are those for which model predictions $M_{c}(I_{s})$ or $M_{c}^{\prime }(E_{s})$ approach 0.5 in our experimental settings of binary classification [41]. Formally, we denote the predictive probability of $I_{s}$ towards the positive class as $M_{c,1}(I_{s})$ and the negative class as $M_{c,0}\left(I_{s}\right)=1-M_{c,1}(I_{s})$.

Least confidence sampling selects the samples whose predictive probabilities $P(I_{s})$ of the most probable class are low. In binary classification, the most probable class is either positive or negative. Given that ${0\leq M}_{c,1}(I_{s})\leq 1$, if $M_{c,1}(I_{s})>0.5$, the most probable class is the positive class, resulting in $P\left(I_{s}\right)=M_{c,1}(I_{s})>0.5$. Conversely, if $M_{c,1}(I_{s})<0.5$, the most probable class is the negative class, and $P\left(I_{s}\right)={1-M}_{c,1}(I_{s})>0.5$. Therefore, the lowest probability occurs when $M_{c,1}(I_{s})=0.5$.

Margin of confidence sampling identifies the samples with small difference between the first and second most probable classes. In binary classification, the difference is expressed as $\left|M_{c,1}\left(I_{s}\right)-M_{c,0}\left(I_{s}\right)\right|=\left|M_{c,1}\left(I_{s}\right)-{1+M}_{c,1}\left(I_{s}\right)\right|$. Clearly, the lowest difference is 0, which is achieved when $M_{c,1}(I_{s})=0.5$.

Entropy sampling [28, 50] chooses the samples with the highest entropy sum of predictive probabilities across all classes. The sum is expressed as ${-M}_{c,1}\left(I_{s}\right)\log\left(M_{c,1}\left(I_{s}\right)\right)-M_{c,0}\left(I_{s}\right)\log\left(M_{c,0}\left(I_{s}\right)\right)={-\;M}_{c,1}\left(I_{s}\right)\log\left(M_{c,1}\left(I_{s}\right)\right)-{(1-M}_{c,1}\left(I_{s}\right))\log\left(1-M_{c,1}\left(I_{s}\right)\right)$ in binary classification. The first derivative of the sum is $-\log(M_{c,1}\left(I_{s}\right)/(1-M_{c,1}\left(I_{s}\right)))$, with a stationary point occurring at $M_{c,1}(I_{s})=0.5$. Consequently, the maximum of the sum is attained when $M_{c,1}(I_{s})=0.5$.

In addition to uncertainty sampling strategies, we implement random sampling, a widely used and well‐established approach [10, 32, 48, 76, 104, 105], to select samples in the subsequent learning stage.

## 3 Experiments

This section begins by presenting a comprehensive overview of the experimental settings, including data sets, AL strategies, DL implementation details, evaluation metrics, and statistical tests. Next, we present the results of various AL strategies applied across previous experimental settings and foundation models developed by supervised and self‐supervised learning. Finally, the statistical test results are analyzed to compare foundation models with their ImageNet counterparts, evaluate pixel‐based classifiers against representation‐based classifiers, examine the relationship between initialization and subsequent learning, and assess the effectiveness of different query strategies: one‐shot initialization versus initialization followed by iterative subsequent learning.

### 3.1 Experimental Settings

#### 3.1.1 Data Sets

To ensure the robustness of our experimental results [106], we employed two data sets featuring diverse population cohorts, sample sizes, and disease categories: the Guangzhou data set [107, 108] and the Pakistan data set [109, 110]. The Guangzhou data set, collected by the Guangzhou Women and Children's Medical Center, comprised 5856 chest radiographs from retrospective cohorts of pediatric patients aged 1–5 years, with 4273 images diagnosed with pneumonia. In contrast, the Pakistan data set was considerably smaller and contained a total of 450 chest radiographs from a local hospital in Pakistan, among which 390 images were diagnosed with COVID‐19. Both data sets were collected after the release of foundation models and were specifically chosen to simulate real‐world scenarios, enabling an assessment of the benefits these models bring to AL. We resized all radiographs from the two data sets into the resolution of 224 × 224 to comply with DL classifiers [111], foundation models [59], and ImageNet counterparts [70].

The data sets were split using an 80/20 ratio for the Guangzhou data set, resulting in 4686 images for training (3419 diseased) and 1170 images for testing (854 diseased). For the Pakistan data set, a 50/50 split was applied, yielding 225 images for both training and testing sets, with 195 diseased images in each set. A larger proportion of samples was allocated to the testing set in the Pakistan data set due to its small sample size, ensuring greater stability in testing. We did not create separate validation sets with diagnostic labels, unlike prior cold‐start AL studies [48, 112] to replicate real‐world cold‐start scenarios where no annotated samples were available at the outset [113]. Additionally, all labels were hidden during the initial sample selection and remained inaccessible until chosen by the query strategy in subsequent learning stages, simulating the cold‐start AL process [51].

#### 3.1.2 Foundation Models and ImageNet Counterparts

TorchXRayVision (TXRV) [114] is an open‐source library developed for chest radiograph analysis, offering a range of representation learning models trained on 950,778 chest radiographs from 13 data sets collected across diverse regions, including the United States, China, Spain, and Vietnam. These models served as feature extractors (representation providers). For input images with a resolution of 224 × 224, TXRV utilizes DenseNet‐121 [115] as its backbone. Notably, TXRV was trained using fully supervised methods rather than self‐supervised approaches. In this study, we used TXRV to compare a domain‐specific supervised model with a general supervised model, specifically the ImageNet pretrained DenseNet‐121 [116].

Robust and Efficient MEDical Imaging with Self‐supervision (REMEDIS) strategy [59] combines supervised pretraining on natural images with contrastive self‐supervised pretraining on chest radiographs. Specifically, it employs the ResNet‐152 architecture [70] with pretrained weights from BiT‐L [117], which were trained on a large‐scale database of natural images (JFT‐300M) [118]. REMEDIS was then trained using the self‐supervised technique of SimCLR [119] on unlabeled medical data sets across five domains: chest radiographs, fundus imaging, digital pathology, mammography, and clinical dermatology. After that, REMEDIS learned generalizable representations that can be paired with a classifier head to map them to domain‐specific labels for downstream tasks. REMEDIS has proven particularly effective for chest radiograph classification [120], and therefore we adopted it as a domain‐specific self‐supervised model for comparison with ImageNet pretrained ResNet‐152. For both TXRV and REMEDIS, we utilized the embeddings from the final layer preceding the classification head as model representations for diverse AL strategies and simplified classifiers.

#### 3.1.3 AL Strategies

For cold‐start initialization, we performed five experiments using budgets ranging from 10 to 50, with an incremental step of 10 samples. Diversity sampling employed classic K‐means clustering, generating a number of clusters equal to the budget, and selecting the sample closest to each cluster centroid. In uncertainty sampling, estimators were trained using inputs of original images and outputs of representations from foundation models or ImageNet counterparts. Estimators then processed each sample 100 times with a dropout activation probability of 0.5 to stably compute the variance of model predictions [121], selecting the samples with the top variance per the allocated budget. The hybrid method integrated diversity and uncertainty strategies by generating a number of clusters equal to the budget and then selecting the sample with the highest variance from each cluster to form the initialization set. Random sampling was simulated 100 times for each initialization budget. For each simulation, the high‐budget group included all samples from the low‐budget group to ensure comparability in downstream analyses.

For subsequent learning, we allocated an initial budget of 10 and a subsequent learning budget of 40 to enable a direct comparison between one‐shot initialization and the full AL strategy with both initialization and subsequent learning. The subsequent learning stage consisted of 4 iterations, each with a budget of 10. Due to the convergence of the three uncertainty strategies, 10 samples with predictive probabilities closest to 0.5 were selected based on the current classifier in each iteration. Random sampling, similar to the initialization, was benchmarked 100 times for comparison. In each iteration of the subsequent learning process, 10 samples were randomly selected from the unlabeled set.

For both initialization and subsequent learning stages, we established the same upper bound of model performance by training classifiers using all available training samples and their expert annotations. This budget was referred to as the “all samples.”

#### 3.1.4 Implementation Details

Two main categories of DL models were developed in this study: one for binary classification tasks and another for uncertainty estimation during the initialization phase. We implemented binary classifiers based on VGG‐11 [111] to distinguish either pneumonia or COVID‐19 in the Guangzhou data set and the Pakistan data set, respectively. VGG‐11 architecture was selected for its extensive use in AL studies [7] and its reliable convergence on small‐sample data sets [122, 123, 124]. In addition to the full VGG‐11 architecture, which used original images as inputs, we also developed simplified models based on previous studies [57, 125]. Specifically, we implemented three‐layer multilayer perceptron (MLP‐3) models with intermediate layers of 512 and 256 neurons, using representations generated by foundation models as inputs [60]. The second category of DL models focused on uncertainty estimation, using the same VGG‐11 architecture but with output layers modified to match the dimensionality of the target representations from foundation models.

For the training of binary classifiers, we utilized a Stochastic Gradient Descent (SGD) optimizer [126] with a learning rate of 1e−3 and a momentum of 0.9. The batch size was fixed at 10, matching the initialization budget. To mitigate the imbalance between major and minor samples [127, 128], a weighted cross‐entropy loss function was applied. Training was conducted for 200 epochs, with a linear scheduler that reduced the learning rate by a factor of 0.5 if no improvement was observed over 10 consecutive epochs. An early stopping criterion was employed if no progress was made over 20 consecutive epochs. For the training of uncertainty estimators, all configurations were kept constant, except for the learning rate, which was adjusted to 1e−4, and the loss function, which was changed to mean squared error to align with the predictive targets of image representations in the continuous space.

Upon the completion of sample annotation queries and binary classifiers' training, the classification performance was assessed on the hold‐out test sets from both the Guangzhou and Pakistan data sets. Following the previous literature [129, 130], the evaluation utilized the area under the receiver operating characteristic curve (AUROC) and the area under the precision‐recall curve (AUPRC) due to their reliability in scenarios involving imbalanced data [131, 132]. Metrics such as accuracy, sensitivity, specificity, positive predictive value, and negative predictive value were excluded due to their vulnerability to instability in the presence of extreme data imbalances [133, 134]. Standard deviations for each metric were estimated using the nonparametric bootstrap method [135]. The study was conducted in PyTorch 1.12.1 and the code has been open access [136] for reproducibility. All experiments were implemented on a Dell Precision 7920 Tower Workstation with an Intel Xeon Silver 4210 CPU and an NVIDIA GeForce RTX 2080 Super GPU.

#### 3.1.5 Statistical Tests

We conducted statistical tests to assess whether significant differences exist in cold‐start AL performance across various configurations [35]. Our first inquiry sought to determine whether domain‐specific foundation models outperform their ImageNet pretrained counterparts. We also investigated whether simplified classifiers based on feature representations could surpass more complex classifiers relying on original image pixels. Additionally, we aimed to establish whether effective initialization contributes to enhanced subsequent learning. Finally, we examined whether a one‐shot initialization can achieve performance comparable to the complete cold‐start AL process, which includes initialization and multiple iterations of subsequent learning; this approach offers greater ease of implementation and user‐friendliness [137, 138]. For the analysis of the relationship between initialization and subsequent learning, we employed the Pearson correlation coefficient [139]. The same correlation test was performed to evaluate the influence of class balance in the initialization samples on model performance in both initialization and subsequent learning. In addressing the other three questions, we utilized the paired *t*‐test [140] to compare the performance of the two competing approaches.

### 3.2 Results

#### 3.2.1 Cold‐Start Initialization

**Figure 2:**
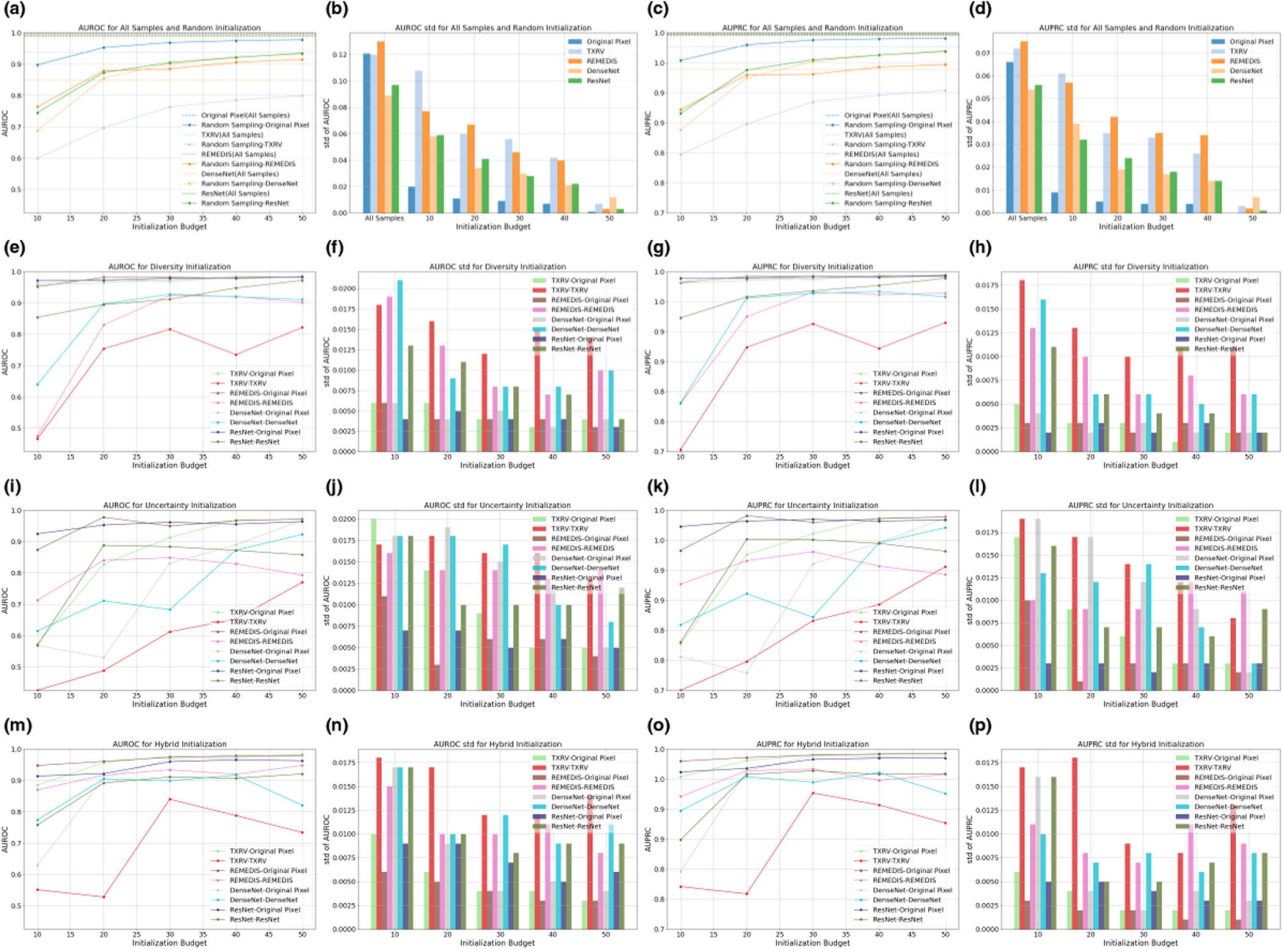
One‐shot initialization performance on the Guangzhou data set. Subgraphs (a–p) present specific information detailed in the following explanations. The first, second, third, and last columns present the mean values of AUROC, the standard deviation of AUROC, the mean values of AUPRC, and the standard deviation of AUPRC, respectively. The first row displays the results for all samples with annotations, the upper bound, and random sampling. The second, third, and final rows show the outcomes for diversity sampling, uncertainty sampling, and hybrid sampling, respectively.

**Figure 3:**
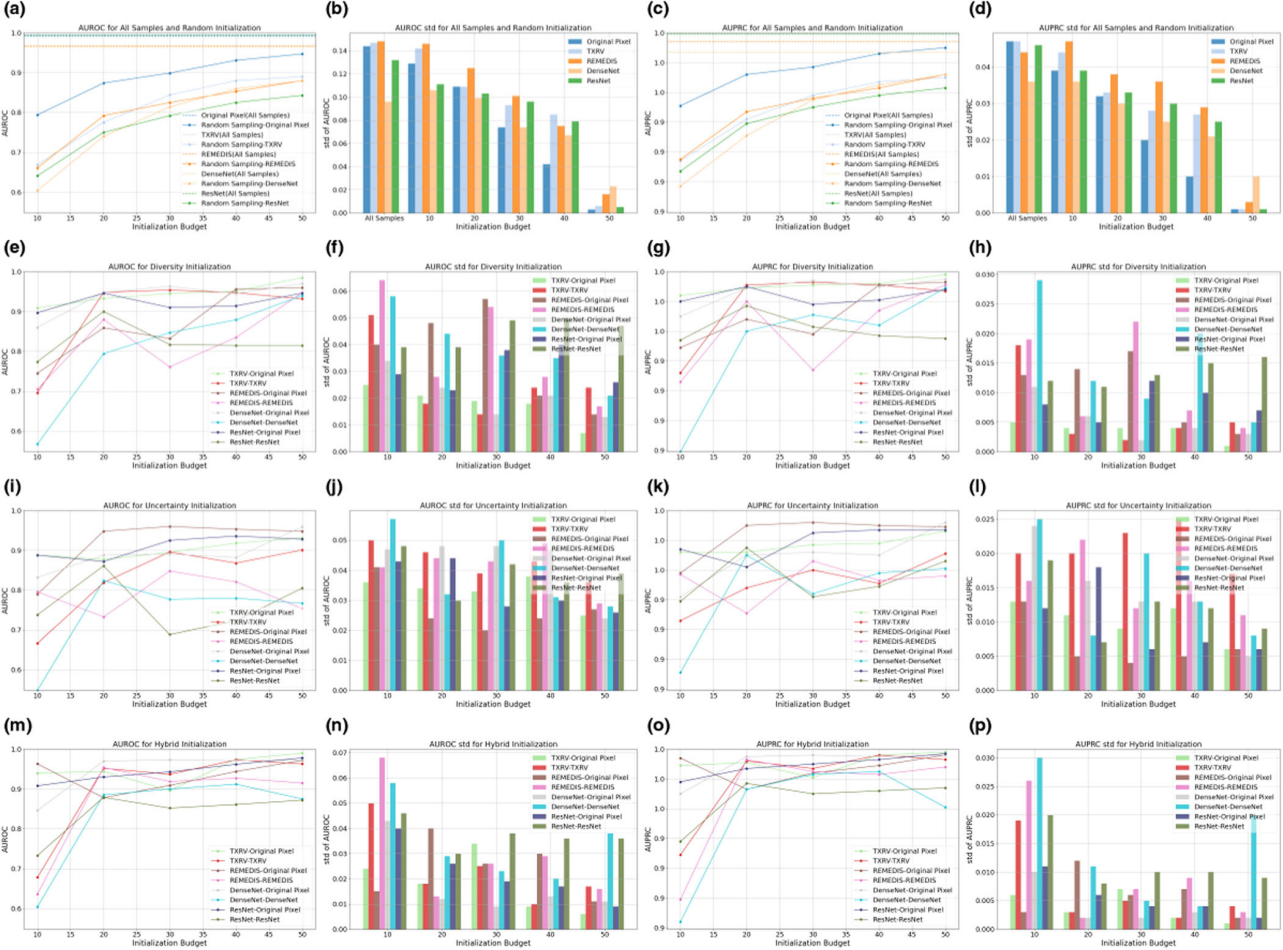
One‐shot initialization performance on the Pakistan data set. Subgraphs (a–p) present specific information detailed in the following explanations. The first, second, third, and last columns present the mean values of AUROC, the standard deviation of AUROC, the mean values of AUPRC, and the standard deviation of AUPRC, respectively. The first row displays the results for all samples with annotations, the upper bound, and random sampling. The second, third, and final rows show the outcomes for diversity sampling, uncertainty sampling, and hybrid sampling, respectively.

Figures 2 and 3 illustrate the AUROC and AUPRC performance of various strategies and model backbones during the cold‐start AL initialization on the Guangzhou and Pakistan data sets, respectively. In the odd‐numbered columns, curve plots depict the mean values of AUROC and AUPRC, whereas the even‐numbered columns present bar plots displaying their standard deviations, calculated via nonparametric bootstrap. The first row in both figures displays two baseline query strategies: all samples and random sampling. The horizontal dashed lines in Figures 2a,c and 3a,c represent the upper bound of classification performance, achieved by training on the full set of samples and annotations. Random sampling was the most common practice in cold‐start initialization, and we compared this method with initialization strategies based on foundation models and ImageNet pretrained counterparts.

The subplots in the second, third, and fourth rows present model performance based on samples selected by diversity, uncertainty, and hybrid sampling, respectively, each applied to the four representation generation models. Using the samples queried by these diverse strategies, we developed both a full VGG‐11 model and a simplified MLP‐3. The MLP‐3, based on representations, consistently underperformed the VGG‐11 model trained on original pixels, suggesting that low‐dimensional representations derived from foundation models and ImageNet counterparts may lose critical information embedded in the original images. For the VGG‐11 classifiers, representation‐based sampling outperformed random sampling in 47 out of 60 scenarios for the Guangzhou data set and 46 out of 60 for the Pakistan data set, indicating that representation‐based strategies reduced annotation requirements while providing superior initializations. Among the three representation‐based strategies, diversity and hybrid sampling achieved the best performance in 16 out of 20 and 14 out of 20 scenarios for the Guangzhou and Pakistan data sets, respectively. This suggested that for data sets with small sample sizes, such as the Pakistan data set, the hybrid method that incorporated both diversity and uncertainty may be preferred. In contrast, for larger data sets, such as the Guangzhou data set, diversity sampling remained competitive. Additionally, across all strategies, as the initialization budget increased, the standard deviation of model performance decreased, demonstrating that a larger sample size not only improved model accuracy but also enhanced prediction robustness. For detailed numeric results, see Tables A1 and A2.

#### 3.2.2 Cold‐Start Subsequent Learning

**Figure 4:**
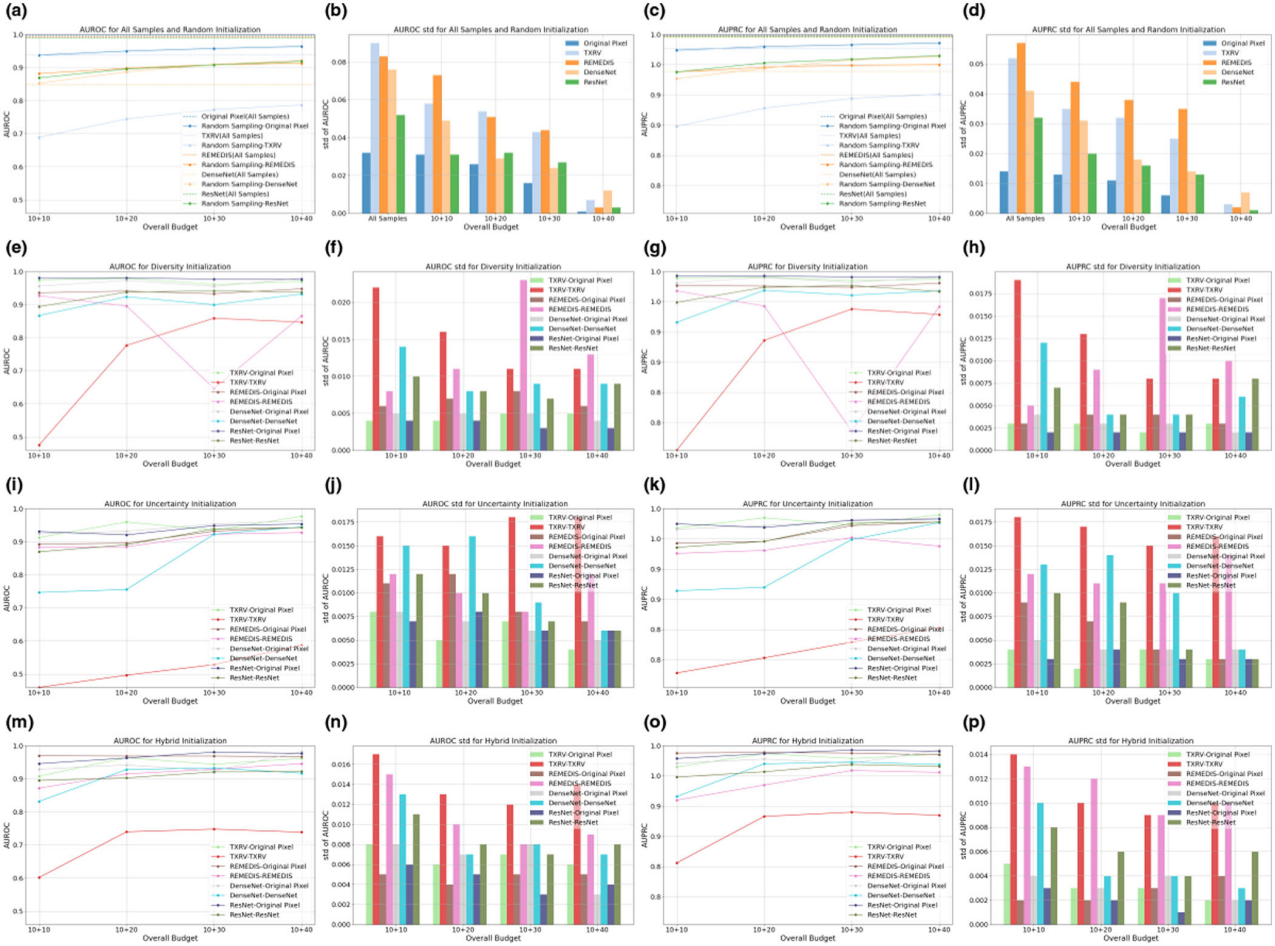
Subsequent learning performance on the Guangzhou data set. Subgraphs (a–p) present specific information detailed in the following explanations. The first, second, third, and last columns present the mean values of AUROC, the standard deviation of AUROC, the mean values of AUPRC, and the standard deviation of AUPRC, respectively. The first row displays the results for all samples with annotations, the upper bound, and random sampling. The second, third, and final rows show the outcomes for diversity sampling, uncertainty sampling, and hybrid sampling, respectively.

**Figure 5:**
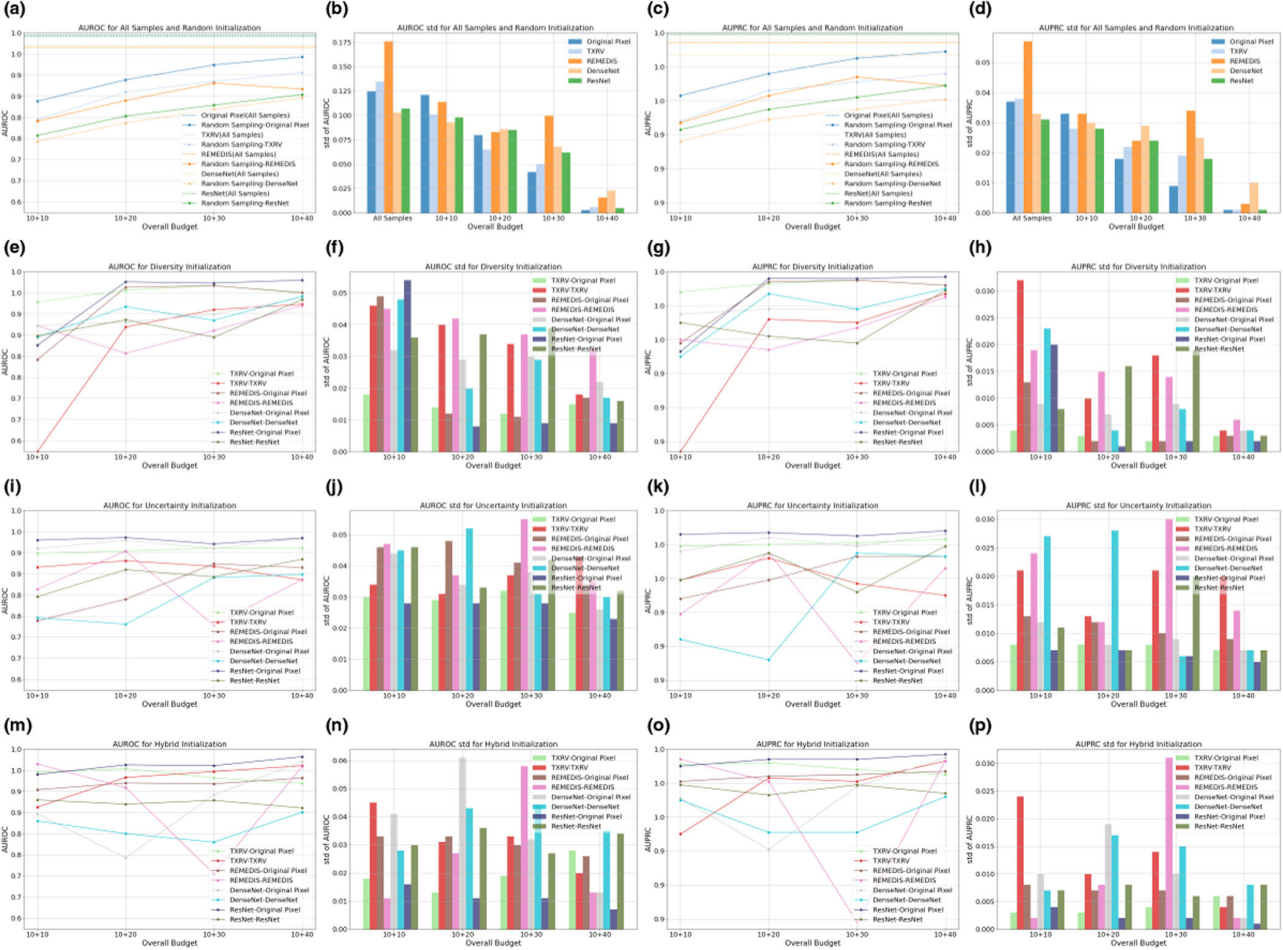
Subsequent learning performance on the Pakistan data set. Subgraphs (a–p) present specific information detailed in the following explanations. The first, second, third, and last columns present the mean values of AUROC, the standard deviation of AUROC, the mean values of AUPRC, and the standard deviation of AUPRC, respectively. The first row displays the results for all samples with annotations, the upper bound, and random sampling. The second, third, and final rows show the outcomes for diversity sampling, uncertainty sampling, and hybrid sampling, respectively.

Based on the classifiers trained on 10 samples selected by different initialization strategies, subsequent learning was performed using uncertainty‐based iterations [23], with 10 samples queried per iteration. Figures 4 and 5 illustrate the classification performance on the Guangzhou and Pakistan data sets, respectively. The arrangement of subplots in Figures 4 and 5 mirrors that of Figures 2 and 3, with the following distinctions: (1) the learning strategy was based solely on classifier uncertainty, and the legend in each subplot indicates the initialization strategy; (2) samples selected under high‐budget conditions included all samples from low budgets as they were consecutive procedures, which was not guaranteed in the initialization stage; and (3) the ***X***‐axis representing the overall budget included both the initialization and subsequent learning phases: For example, a budget of 10 + 20 denoted an initialization budget of 10 samples, followed by an additional budget of 20 samples for the subsequent learning.

As annotation budgets increased, VGG‐11 performance initially improved over multiple iterations before quickly converging, with additional samples yielding only marginal gains. This phenomenon was attributed to the relative simplicity of the two binary classification tasks compared to more complex clinical tasks, such as low‐contrast lesion segmentation [141, 142, 143], as demonstrated by the strong classification performance before subsequent learning. Although some performance improvement to the upper bound in Figures 4a,c and 5a,c remained possible, achieving this would require approximately 100 times more annotations for the Guangzhou data set and 10 times more for the Pakistan data set. This underscored the effectiveness of AL in balancing annotation costs with model performance. Compared with VGG‐11 models, the representation‐based MLP‐3 classifiers were inferior, aligning with previous initialization results. Additionally, MLP‐3 classifiers exhibited the risk of overfitting in Figures 4e,g and 5i,k,m,o: their predictive performance declined when more samples were added to the labeled training set. For detailed numeric results, see Tables A3 and A4.

Models initialized with diversity and hybrid sampling consistently outperformed uncertainty sampling, achieving the highest performance in 8 and 7 out of 16 learning scenarios for the Guangzhou data set, and 8 and 5 out of 16 scenarios for the Pakistan data set. This consistent outperformance of diversity and hybrid sampling highlighted the benefit of effective initialization for both the initialization and subsequent learning stages. In the next subsection, we will extend these observational findings with rigorous statistical tests. Specifically, we would compare representations from foundation models and ImageNet counterparts, evaluate different classification backbones, explore the relationship between initialization and subsequent learning, and contrast one‐shot initialization with the complete AL process.

#### 3.2.3 Comparative Study on Classification Backbones

The primary question addressed in this study was whether foundation models designed for chest radiograph analysis outperformed their ImageNet counterparts pretrained on images from the natural domain. We conducted a paired *t*‐test to compare their performance during both the initialization and subsequent learning stages using the Guangzhou and Pakistan data sets. The null hypothesis posited that the performance of foundation models and ImageNet counterparts would be statistically identical, whereas the alternative hypothesis suggested that the performance of foundation models is superior to that of ImageNet counterparts. As shown in Table 1, foundation models outperformed their ImageNet counterparts in only two out of eight experiments. Consequently, in the context of cold‐start AL, foundation models failed to meet our expectations as generalist models.

**Table 1:** Paired *t*‐test between classifiers initialized based on foundation models and ImageNet pretrained counterparts.

Data set	Foundation model	AL stage	*p*‐value of AUROC	*p*‐value of AUPRC
Guangzhou data set	TXRV	Cold‐start initialization	2.74e−2*	2.89e−2*
		Subsequent learning	3.10e−1	1.57e−1
	REMEDIS	Cold‐start initialization	1.59e−1	2.94e−1
		Subsequent learning	9.96e−1	9.97e−1
Pakistan data set	TXRV	Cold‐start initialization	2.59e−1	9.34e−2
		Subsequent learning	4.80e−2*	2.99e−2*
	REMEDIS	Cold‐start initialization	8.47e−1	7.54e−1
		Subsequent learning	9.98e−1	9.96e−1

* The *p*‐value is less than 0.05, demonstrating statistical significance at a confidence level of 95%.

Another objective of the foundation model was to generate representations that could be directly utilized as input features, thereby facilitating lightweight classification backbones, such as MLP, to achieve high‐fidelity predictions with reduced computational costs [57, 59, 114]. To assess this, we compared the performance of VGG‐11 with that of the lightweight MLP‐3 [57, 125]. The null hypothesis posited that the performance of MLP‐3 using generated representations was equivalent to that of VGG‐11 using original pixel data, whereas the alternative hypothesis proposed that the performance of MLP‐3 was inferior to that of VGG‐11. Table 2 illustrates that MLP‐3 statistically significantly underperformed VGG‐11 in seven out of eight scenarios.

**Table 2:** Paired *t*‐test between MLP‐3 using representations and VGG‐11 using original pixels in cold‐start initialization.

Data set	Representation source	AL stage	*p*‐value of AUROC	*p*‐value of AUPRC
Guangzhou data set	TXRV	Cold‐start initialization	3.62e−10*	3.52e−9*
		Subsequent learning	2.30e−7*	8.49e−7*
	REMEDIS	Cold‐start initialization	3.35e−5*	1.81e−6*
		Subsequent learning	2.35e−3*	1.83e−3*
Pakistan data set	TXRV	Cold‐start initialization	1.98e−3*	1.88e−4*
		Subsequent learning	9.99e−3*	1.33e−3*
	REMEDIS	Cold‐start initialization	3.83e−4*	6.29e−4*
		Subsequent learning	7.91e−2	1.42e−2*

* The *p*‐value is less than 0.05, demonstrating statistical significance at a confidence level of 95%.

We identified that representation‐based strategies outperformed default random sampling during the initialization stage. However, the extent to which these benefits extend to subsequent learning stages remained inadequately explored. To investigate this, we calculated the Pearson correlation coefficient of the AUROC and the AUPRC between model performance in the initialization stage and the subsequent learning stage. Our null hypothesis was that the correlation coefficient between the performance of cold‐start initialization and subsequent learning did not significantly deviate from zero, whereas the alternative hypothesis asserted that this correlation was significantly greater than zero. As shown in Table 3, model performance during the initialization stage was positively correlated with performance in the subsequent learning stage, suggesting that researchers should pay more attention to effective initialization strategies instead of using random sampling as a default [144].

**Table 3:** Pearson correlation coefficient between cold‐start initialization and subsequent learning. Classifiers were developed using the same architecture of VGG‐11 and original pixels.

Data set	Overall budget	*p*‐value of AUROC	*p*‐value of AUPRC
Guangzhou data set	10 + 10	2.69e−5*	2.13e−5*
	10 + 20	4.03e−5*	8.33e−5*
	10 + 30	2.56e−5*	7.98e−4*
	10 + 40	2.05e−4*	5.52e−4*
Pakistan data set	10 + 10	3.66e−3*	4.92e−3*
	10 + 20	1.19e−3*	1.82e−3*
	10 + 30	1.23e−3*	6.84e−3*
	10 + 40	2.89e−3*	7.61e−3*

* The *p*‐value is less than 0.05, demonstrating statistical significance at a confidence level of 95%.

Another question we sought to address was whether one‐shot initialization identified samples capable of training models with performance comparable to those selected through both initialization and iterative learning stages. Consistent with the first and second statistical tests, we conducted a paired *t*‐test between the two approaches using an equivalent overall budget. The null hypothesis posited that the average performance of classifiers utilizing one‐shot initialization was identical to that of classifiers employing a full AL cycle of both initialization and subsequent learning. Conversely, the alternative hypothesis asserted that the performance of classifiers using one‐shot initialization was inferior to that of classifiers employing the integrated approach. As presented in Table 4, all *p*‐values exceeded 0.05, indicating that one‐shot initialization was comparable to the complete AL cycle in the medical task of chest radiograph classification.

**Table 4:** Paired *t*‐test between classifiers developed using one‐shot initialization and complete AL cycle.

Data set	Overall budget		*p*‐value of AUROC	*p*‐value of AUPRC
	Initialization‐only	Initialization + subsequent learning		
Guangzhou data set	20	10 + 10	4.38e−1	5.48e−1
	30	10 + 20	8.04e−1	7.99e−1
	40	10 + 30	8.08e−1	7.81e−1
	50	10 + 40	5.41e−1	5.74e−1
Pakistan data set	20	10 + 10	9.74e−1	9.84e−1
	30	10 + 20	4.56e−1	5.15e−1
	40	10 + 30	8.94e−1	9.36e−1
	50	10 + 40	5.38e−1	4.58e−1

Finally, we investigated whether model performance was influenced by the class balance of the initialization samples, specifically testing the hypothesis that a balanced class distribution could enhance performance. The null hypothesis posited no significant correlation deviating from zero between the minority class proportion in the initialization samples and the classifier's performance during cold‐start initialization or subsequent learning. In contrast, the alternative hypothesis suggested that this correlation was significantly greater than zero. As shown in Table 5, no statistically significant correlation was observed in both stages. Interestingly, a U‐shaped trend in *p*‐values was observed during both stages of the Guangzhou data set and the cold‐start initialization of the Pakistan data set, indicating that the class balance was more strongly correlated with performance at intermediate budget levels compared to low or high budgets.

**Table 5:** Pearson correlation coefficient between minority class proportion and model performance in different AL stages. Classifiers were developed using the same architecture of VGG‐11 and original pixels.

Data set	AL stage	Overall budget	*p*‐value of AUROC	*p*‐value of AUPRC
Guangzhou data set	Cold‐start initialization	10	0.283	0.234
		20	0.099	0.094
		30	0.056	0.052
		40	0.063	0.055
		50	0.453	0.530
	Subsequent learning	10 + 10	0.240	0.102
		10 + 20	0.089	0.057
		10 + 30	0.079	0.069
		10 + 40	0.300	0.316
Pakistan data set	Cold‐start initialization	10	0.196	0.535
		20	0.297	0.506
		30	0.131	0.168
		40	0.816	0.813
		50	0.793	0.753
	Subsequent learning	10 + 10	0.080	0.098
		10 + 20	0.186	0.210
		10 + 30	0.667	0.658
		10 + 40	0.809	0.867

## 4 Discussion

In this study, we conducted a quantitative analysis to evaluate the performance of domain‐specific pretrained models compared to their ImageNet counterparts during both the initialization and learning stages of cold‐start AL. Unlike foundation models in natural language processing [145, 146, 147], our findings reveal a notable disparity in the efficiency of pretrained models within the domain of medical imaging [148]. In most experiments, models pretrained on chest radiographs, whether through supervised or self‐supervised learning, did not surpass those pretrained on natural images in selecting informative samples for cold‐start AL. Also, the representation did not improve the performance of a simplified model based on MLP architectures, contrary to researchers' expectations that it would surpass the performance of a more complex model using original images as inputs. Additionally, the class balance of initialization samples did not consistently exhibit a positive correlation with model performance across varying budgets in AL initialization and subsequent learning.

The relative inefficiency of domain‐specific pretrained models compared to ImageNet‐trained models can be attributed to several factors. In general, when domain‐specific models are trained on a limited number of samples, their generalization capabilities are often inferior to those of ImageNet‐trained models, primarily due to differences in sample and class diversity [149]. However, in our experiments, both domain‐specific models were trained on data sets of comparable size to ImageNet. Beyond data scale, model architecture also influences the representation learning capacity of pretrained models [150, 151]. In this study, we standardized the architecture across domain‐specific and ImageNet‐trained models, ensuring that model architecture did not influence the comparison outcomes. We hypothesize that the inefficiency raised because the latent features in the two data sets may not be fundamentally complex, as evidenced by the rapid model convergence with only a few annotated samples. Thus, despite chest radiographs being visually distinct from general domain images, the low‐ to mid‐level features learned from ImageNet appear sufficient for effectively discriminating between different images in this context [152, 153, 154].

A recent study by Huix et al. [145] also reports similar experimental results. They evaluated five vision transformer‐based foundation models: SAM [155], SEEM [156], DINOv2 [157], CLIP [158], and BLIP [159], across four well‐established medical imaging data sets. All five models employ transformer‐based architectures, allowing for direct comparison with the baseline ImageNet pretrained vision transformer. The results revealed that only one model, DINOv2, consistently outperformed the ImageNet pretrained counterpart in four comparative experimental configurations, including whether a linear head or a complex DeiT [160] was used on top of the foundation models, and whether the foundation model parameters were frozen or not. Similar to our findings, the frozen foundation models with a linear head performed worse than those with DeiT, a more complex architecture. Interestingly, when the foundation model parameters were fine‐tuned using target data, the linear head outperformed the transformer, a finding that merits further investigation. Although their work focused on comparing general domain foundation models with ImageNet‐trained models in diverse medical imaging tasks, our study addresses a gap by further assessing whether models specifically designed for chest radiograph analysis can outperform ImageNet‐trained counterparts in tasks within the target domains.

This study also uncovered inspiring findings. First, compared to the commonly used random sampling strategy, which has demonstrated decent performance in prior work [74, 161, 162], both chest radiograph pretrained models and their ImageNet counterparts led to improved performance. This suggests that representation‐based initialization may be a superior alternative to random sampling for future AL applications, potentially achieving classifier performance comparable to models trained on fully annotated data sets [141, 142]. The advantages of a robust initialization were further supported by a statistically significant positive correlation between initial model performance and subsequent learning outcomes.

Second, we found that one‐shot initialization performed on par with complete AL across both the Guangzhou and Pakistan data sets. This approach alleviates the need for repeated experts' involvement during AL iterations, enabling continuous training of DL models without delays caused by awaiting new sample annotations [137, 138]. Similar one‐shot initialization strategies, such as representative annotation, have also been explored in recent studies [72]. Specifically, it has two components to select samples: the first component uses autoencoder [163], variational autoencoder [164], or generative adversarial networks [165] to learn efficient data representation in an unsupervised manner. Based on these clustering‐friendly representations, the second component uses agglomerative clustering and applies the greedy max‐cover strategy to select images from each cluster. In 2D gland segmentation, the one‐shot initialization method demonstrated performance comparable to state‐of‐the‐art iterative approaches while remarkably reducing experts' waiting times. This time‐saving advantage was even more pronounced in 3D segmentation of myocardium and great vessels. Jin et al. [138] proposed a one‐shot AL method that integrates contrastive learning with diversity sampling. Their approach demonstrated superior performance compared to random sampling and two iterative AL strategies of Bayesian sample query [166] and core‐set [167] in skin lesion segmentation, remote sensing image segmentation, and chest x‐ray segmentation. The two preceding one‐shot AL methods rely on informative representations, highlighting the potential of exploring domain‐specific foundation models as representation providers.

Third, although no statistically significant correlation was observed between the sample balance ratio and model performance, a U‐shaped trend in *p*‐values suggests that the class balance is more strongly associated with performance at intermediate budget levels than at low or high budgets. We propose that this phenomenon arises because, in low‐budget scenarios, the data set's balance ratio exerts minimal influence on model performance, as the limited number of training samples constrains the achievable upper bound of model performance. Conversely, in high‐budget scenarios, the abundant training samples ensure the lower bound of model performance, thereby limiting the observable impact of the balance ratio. In both cases, model performance is confined within a relatively narrow range, making it challenging to detect significant correlations.

Our study has limitations that warrant future investigation. First, this study exclusively examined the use of supervised learning based on labeled samples. Future researchers may explore the augmentation of labeled samples [168, 169] or training strategy of semisupervision [54, 112] or metalearning [170, 171] to further upgrade model performance without additional annotation burden [29]. From the data augmentation perspective, Shi et al. proposed to stitch four intraclass images together and resize them to the same size as the original image to unleash the potential value of limited annotated samples [31]. From both empirical improvements in AL performance and theoretical distribution similarity in high‐level semantic space, they validated the positive impact of data augmentation towards AL [172]. Beyond aggregation of existing samples in the pixel space [173], Mahapatra et al. employed generative adversarial networks [165] to synthesize realistic chest radiographs from a limited set of anatomy annotations [174]. By incorporating these generated samples and associated annotations into the training set, they achieved a substantial improvement in model accuracy. From the semisupervised view, Bai et al. proposed to combine expert‐annotating labels with model‐predicting pseudo labels to boost model performance [175]. To eliminate the training instability caused by pseudo labels, they designed a noise filter to filter pseudo labels with low fidelity, avoiding the improvement brought by informative pseudo labels being impaired by noisy ones [176]. Metalearning is another direction to improve DL performance using auxiliary tasks to generate a robust model that converges to the target task with minimal labeled samples [177]. Yuan et al. designed a training strategy that combines metalearning with AL, including two phases where the first phase aims to pretrain a metalearner possessing sensitive perceptron on the target data domain and the second phase is to select samples with the highest uncertainty on the target task [90]. The main difference between the foundation model and metamodel is that the generation mechanism is based on self‐supervised learning or auxiliary tasks‐based supervised learning and the integration of these two techniques has been investigated by recent studies [178, 179].

Second, our pipeline for cold‐start AL was designed with modular components, and substituting the current techniques with alternative methods would enhance the credibility of our current findings. Foundation models can be replaced with momentum contrast for chest x‐rays [180, 181] or in‐house developed self‐supervised models [72, 182]. Sampling strategies can also be extended to advanced techniques. For example, the current diversity sampling used K‐means as the backbone and designated the sample closest to cluster centers as the representative one. Moving forward, we will include refined metrics of representativeness such as information density, which calculates the similarity between embeddings of a particular sample and others within the same cluster [23]. K‐means can also be substituted with alternative methods such as BIRCH [183], which empirical evidence suggests is more robust against noisy data and imbalanced labels [23]. Similarly, the hybrid sampling strategy follows a static combination of representativeness and uncertainty while some dynamic reweighting combinations may achieve superior performance [27]. Furthermore, the static strategies can be enhanced with reinforcement learning policies such as multiarmed bandit [184] or actor‐critic method [30] to actively switch different sampling strategies based on the state of classifiers and the current environment [185]. Additionally, we evaluated the performance of cold‐start AL within a limited set of configurations, comprising one DL backbone, one imaging modality, two close‐set binary classification targets [186], and no consideration of real labeling time. Future endeavors may encompass alternative DL backbones including vision transformer [187], additional imaging modalities such as positron emission tomography [188], different targets like open‐set classification [28] or lesion segmentation, and comparison at both levels of sample numbers and overall annotation time [32] for a more thorough comparison [189, 190]. These comprehensive experiments would further substantiate the findings of this study regarding the application of foundation models in AL.

Last, our study represents an initial attempt to leverage domain‐specific foundation models in AL and highlights promising avenues for future research in both foundation models and AL strategies. For foundation models, they did not exhibit superior generalization capabilities compared to general pretrained models, highlighting the need for further refinement to achieve their intended objective of versatile performance across diverse tasks [191]. Future research could explore the integration of medical knowledge or the adoption of a federated learning framework [192, 193, 194] to construct substantially larger training data sets, thereby enhancing model performance in accordance with scaling laws [87, 195]. For AL strategies, foundation models can serve as providers of representations. A low‐hanging fruit is to integrate foundation models with strategies that require representations at specific stages of AL or to directly replace DL backbones in target tasks with foundation models, thereby exploring whether AL performance can be enhanced. A more ambitious direction is to exploit the potential of foundation models across different modalities for joint AL. On the one hand, joint AL can involve multiple modalities within medical imaging, such as magnetic resonance imaging, computed tomography, and positron emission tomography [196, 197, 198]. On the other hand, it can involve combining a medical imaging modality with another modality, such as chest radiographs and radiological reports [199, 200, 201, 202, 203].

## 5 Conclusion

Pretraining has been a cornerstone of DL‐based chest radiograph analysis, yet it remains unresolved whether domain‐specific pretraining outperforms general domain pretraining in the context of cold‐start AL. In this study, we demonstrated the inefficiency of domain‐specific foundation models compared to general pretrained ImageNet models for two binary classification tasks. Despite this, initialization methods based on both models significantly outperformed random sampling, the default method for cold‐start AL initialization. Furthermore, we uncovered a positive correlation between different stages of cold‐start AL and found comparable performance between one‐shot initialization and full AL processes. In addition, the influence of class balance in the initialization samples on subsequent learning outcomes warrants careful consideration, particularly in middle‐budget scenarios. We anticipate that this study will inspire researchers to enhance pretraining for generalist medical artificial intelligence and explore novel AL methods based on various pretrained models.

## Author Contributions

**Han Yuan:** conceptualization (lead), data curation (lead), formal analysis (lead), investigation (lead), methodology (lead), software (lead), validation (lead), visualization (lead), writing – original draft (lead), writing – review and editing (lead). **Mingcheng Zhu:** formal analysis (supporting), visualization (lead), writing – review and editing (supporting). **Rui Yang:** formal analysis (supporting), validation (lead), writing – review and editing (supporting). **Han Liu:** investigation (supporting), methodology (supporting), writing – review and editing (supporting). **Irene Li:** formal analysis (supporting), resources (lead), writing – review and editing (supporting). **Chuan Hong:** formal analysis (supporting), investigation (supporting), methodology (supporting), project administration (lead), resources (lead), writing – review and editing (supporting).

## Ethics Statement

Ethics approval was not required for this study as it utilized retrospective data sets that are publicly accessible. Researchers seeking access to the original data should request permission from the data owners and comply with their established protocols on data privacy and confidentiality.

## Conflicts of Interest

The authors declare no conflicts of interest.

## Data Availability

The data sets used in this study are available on https://data.mendeley.com/datasets/rscbjbr9sj/3 and https://www.kaggle.com/datasets/muhammadshahbazkhan/covid19-pakistani-patients-xray-image-dataset.

## 1

**Table A1:** Initialization performance of random sampling, diversity, uncertainty, and hybrid methods on the Guangzhou data set.

Initialization budget	Foundation model	Initialization method	Model input	AUROC	AUPRC
All samples	/	/	Original pixel	0.998 (0.001)	0.999 (0.000)
			TXRV	0.938 (0.007)	0.977 (0.003)
			REMEDIS	0.993 (0.003)	0.997 (0.002)
			DenseNet	0.848 (0.012)	0.939 (0.007)
			ResNet	0.990 (0.003)	0.996 (0.001)
10	/	Random	Original pixel	0.898 (0.121)	0.954 (0.066)
			TXRV	0.599 (0.120)	0.797 (0.072)
			REMEDIS	0.764 (0.130)	0.872 (0.075)
			DenseNet	0.688 (0.089)	0.838 (0.054)
			ResNet	0.745 (0.097)	0.866 (0.056)
	TXRV	Diversity	Original pixel	0.958 (0.006)	0.981 (0.005)
			TXRV	0.466 (0.018)	0.703 (0.018)
		Uncertainty	Original pixel	0.573 (0.020)	0.777 (0.017)
			TXRV	0.425 (0.017)	0.700 (0.019)
		Hybrid	Original pixel	0.885 (0.010)	0.954 (0.006)
			TXRV	0.551 (0.018)	0.771 (0.017)
	REMEDIS	Diversity	Original pixel	0.952 (0.006)	0.982 (0.003)
			REMEDIS	0.475 (0.019)	0.780 (0.013)
		Uncertainty	Original pixel	0.874 (0.011)	0.933 (0.010)
			REMEDIS	0.713 (0.016)	0.877 (0.010)
		Hybrid	Original pixel	0.948 (0.006)	0.980 (0.003)
			REMEDIS	0.870 (0.015)	0.921 (0.011)
	DenseNet	Diversity	Original pixel	0.963 (0.006)	0.983 (0.004)
			DenseNet	0.640 (0.021)	0.781 (0.016)
		Uncertainty	Original pixel	0.568 (0.018)	0.756 (0.019)
			DenseNet	0.615 (0.018)	0.809 (0.013)
		Hybrid	Original pixel	0.629 (0.017)	0.796 (0.016)
			DenseNet	0.774 (0.017)	0.897 (0.010)
	ResNet	Diversity	Original pixel	0.972 (0.004)	0.989 (0.002)
			ResNet	0.854 (0.013)	0.923 (0.011)
		Uncertainty	Original pixel	0.925 (0.007)	0.973 (0.003)
			ResNet	0.569 (0.018)	0.780 (0.016)
		Hybrid	Original pixel	0.914 (0.009)	0.962 (0.005)
			ResNet	0.758 (0.017)	0.849 (0.016)
20	/	Random	Original pixel	0.953 (0.020)	0.980 (0.009)
			TXRV	0.697 (0.108)	0.848 (0.061)
			REMEDIS	0.879 (0.077)	0.930 (0.057)
			DenseNet	0.856 (0.058)	0.925 (0.039)
			ResNet	0.873 (0.059)	0.938 (0.032)
	TXRV	Diversity	Original pixel	0.964 (0.006)	0.985 (0.003)
			TXRV	0.754 (0.016)	0.874 (0.013)
		Uncertainty	Original pixel	0.828 (0.014)	0.926 (0.009)
			TXRV	0.488 (0.018)	0.748 (0.017)
		Hybrid	Original pixel	0.957 (0.006)	0.981 (0.004)
			TXRV	0.528 (0.017)	0.759 (0.018)
	REMEDIS	Diversity	Original pixel	0.982 (0.004)	0.992 (0.003)
			REMEDIS	0.830 (0.013)	0.925 (0.010)
		Uncertainty	Original pixel	0.978 (0.003)	0.991 (0.001)
			REMEDIS	0.840 (0.014)	0.916 (0.009)
		Hybrid	Original pixel	0.961 (0.005)	0.986 (0.002)
			REMEDIS	0.917 (0.010)	0.964 (0.008)
	DenseNet	Diversity	Original pixel	0.977 (0.004)	0.991 (0.002)
			DenseNet	0.897 (0.009)	0.956 (0.006)
		Uncertainty	Original pixel	0.530 (0.019)	0.729 (0.017)
			DenseNet	0.711 (0.018)	0.861 (0.012)
		Hybrid	Original pixel	0.906 (0.009)	0.964 (0.004)
			DenseNet	0.905 (0.010)	0.954 (0.007)
	ResNet	Diversity	Original pixel	0.972 (0.005)	0.989 (0.003)
			ResNet	0.895 (0.011)	0.958 (0.006)
		Uncertainty	Original pixel	0.953 (0.007)	0.982 (0.003)
			ResNet	0.888 (0.010)	0.952 (0.007)
		Hybrid	Original pixel	0.922 (0.009)	0.968 (0.005)
			ResNet	0.892 (0.010)	0.958 (0.005)
30	/	Random	Original pixel	0.969 (0.011)	0.988 (0.005)
			TXRV	0.763 (0.060)	0.885 (0.035)
			REMEDIS	0.885 (0.067)	0.931 (0.042)
			DenseNet	0.901 (0.034)	0.952 (0.019)
			ResNet	0.905 (0.041)	0.955 (0.024)
	TXRV	Diversity	Original pixel	0.973 (0.004)	0.989 (0.003)
			TXRV	0.816 (0.012)	0.913 (0.010)
		Uncertainty	Original pixel	0.913 (0.009)	0.961 (0.006)
			TXRV	0.612 (0.016)	0.816 (0.014)
		Hybrid	Original pixel	0.977 (0.004)	0.991 (0.002)
			TXRV	0.840 (0.012)	0.927 (0.009)
	REMEDIS	Diversity	Original pixel	0.982 (0.004)	0.992 (0.002)
			REMEDIS	0.924 (0.008)	0.966 (0.006)
		Uncertainty	Original pixel	0.950 (0.006)	0.980 (0.003)
			REMEDIS	0.849 (0.014)	0.931 (0.009)
		Hybrid	Original pixel	0.974 (0.004)	0.990 (0.002)
			REMEDIS	0.934 (0.010)	0.967 (0.007)
	DenseNet	Diversity	Original pixel	0.968 (0.005)	0.987 (0.003)
			DenseNet	0.928 (0.008)	0.964 (0.006)
		Uncertainty	Original pixel	0.831 (0.015)	0.911 (0.012)
			DenseNet	0.683 (0.017)	0.822 (0.014)
		Hybrid	Original pixel	0.969 (0.004)	0.989 (0.002)
			DenseNet	0.899 (0.012)	0.945 (0.008)
	ResNet	Diversity	Original pixel	0.979 (0.004)	0.992 (0.002)
			ResNet	0.912 (0.008)	0.968 (0.004)
		Uncertainty	Original pixel	0.962 (0.005)	0.985 (0.002)
			ResNet	0.884 (0.010)	0.951 (0.007)
		Hybrid	Original pixel	0.960 (0.007)	0.983 (0.004)
			ResNet	0.911 (0.008)	0.964 (0.005)
40	/	Random	Original pixel	0.975 (0.009)	0.990 (0.004)
			TXRV	0.785 (0.056)	0.896 (0.033)
			REMEDIS	0.906 (0.046)	0.943 (0.035)
			DenseNet	0.921 (0.030)	0.963 (0.017)
			ResNet	0.922 (0.028)	0.963 (0.018)
	TXRV	Diversity	Original pixel	0.985 (0.003)	0.994 (0.001)
			TXRV	0.735 (0.015)	0.872 (0.011)
		Uncertainty	Original pixel	0.969 (0.005)	0.987 (0.003)
			TXRV	0.653 (0.016)	0.843 (0.012)
		Hybrid	Original pixel	0.976 (0.004)	0.991 (0.002)
			TXRV	0.788 (0.012)	0.907 (0.008)
	REMEDIS	Diversity	Original pixel	0.979 (0.004)	0.992 (0.003)
			REMEDIS	0.921 (0.007)	0.962 (0.008)
		Uncertainty	Original pixel	0.967 (0.006)	0.986 (0.003)
			REMEDIS	0.829 (0.013)	0.907 (0.012)
		Hybrid	Original pixel	0.979 (0.003)	0.992 (0.001)
			REMEDIS	0.920 (0.011)	0.948 (0.011)
	DenseNet	Diversity	Original pixel	0.983 (0.003)	0.993 (0.002)
			DenseNet	0.920 (0.008)	0.967 (0.005)
		Uncertainty	Original pixel	0.890 (0.012)	0.946 (0.009)
			DenseNet	0.873 (0.010)	0.946 (0.007)
		Hybrid	Original pixel	0.973 (0.005)	0.987 (0.004)
			DenseNet	0.917 (0.009)	0.961 (0.006)
	ResNet	Diversity	Original pixel	0.980 (0.004)	0.991 (0.003)
			ResNet	0.948 (0.007)	0.977 (0.004)
		Uncertainty	Original pixel	0.956 (0.006)	0.982 (0.003)
			ResNet	0.872 (0.010)	0.945 (0.006)
		Hybrid	Original pixel	0.966 (0.005)	0.985 (0.003)
			ResNet	0.907 (0.009)	0.958 (0.007)
50	/	Random	Original pixel	0.978 (0.007)	0.991 (0.004)
			TXRV	0.799 (0.042)	0.904 (0.026)
			REMEDIS	0.915 (0.040)	0.947 (0.034)
			DenseNet	0.936 (0.021)	0.970 (0.014)
			ResNet	0.934 (0.022)	0.969 (0.014)
	TXRV	Diversity	Original pixel	0.982 (0.004)	0.993 (0.002)
			TXRV	0.822 (0.014)	0.915 (0.011)
		Uncertainty	Original pixel	0.971 (0.005)	0.989 (0.003)
			TXRV	0.770 (0.013)	0.906 (0.008)
		Hybrid	Original pixel	0.984 (0.003)	0.993 (0.002)
			TXRV	0.734 (0.014)	0.877 (0.013)
	REMEDIS	Diversity	Original pixel	0.983 (0.003)	0.994 (0.002)
			REMEDIS	0.902 (0.010)	0.964 (0.006)
		Uncertainty	Original pixel	0.972 (0.004)	0.989 (0.002)
			REMEDIS	0.793 (0.014)	0.893 (0.011)
		Hybrid	Original pixel	0.980 (0.003)	0.993 (0.001)
			REMEDIS	0.948 (0.008)	0.958 (0.009)
	DenseNet	Diversity	Original pixel	0.981 (0.004)	0.992 (0.002)
			DenseNet	0.911 (0.010)	0.958 (0.006)
		Uncertainty	Original pixel	0.968 (0.005)	0.987 (0.002)
			DenseNet	0.923 (0.008)	0.971 (0.003)
		Hybrid	Original pixel	0.978 (0.004)	0.990 (0.003)
			DenseNet	0.821 (0.011)	0.926 (0.008)
	ResNet	Diversity	Original pixel	0.984 (0.003)	0.993 (0.002)
			ResNet	0.972 (0.004)	0.989 (0.002)
		Uncertainty	Original pixel	0.964 (0.005)	0.984 (0.003)
			ResNet	0.858 (0.012)	0.932 (0.009)
		Hybrid	Original pixel	0.963 (0.006)	0.985 (0.003)
			ResNet	0.921 (0.009)	0.959 (0.008)

**Table A2:** Initialization performance of random sampling, diversity, uncertainty, and hybrid methods on the Pakistan data set.

Initialization budget	Foundation model	Initialization method	Model input	AUROC	AUPRC
All samples	/	/	Original pixel	0.994 (0.003)	0.999 (0.001)
			TXRV	0.991 (0.006)	0.999 (0.001)
			REMEDIS	0.968 (0.016)	0.994 (0.003)
			DenseNet	0.964 (0.023)	0.987 (0.010)
			ResNet	0.993 (0.005)	0.999 (0.001)
10	/	Random	Original pixel	0.794 (0.144)	0.951 (0.047)
			TXRV	0.669 (0.147)	0.914 (0.047)
			REMEDIS	0.661 (0.148)	0.915 (0.044)
			DenseNet	0.604 (0.096)	0.897 (0.036)
			ResNet	0.641 (0.132)	0.907 (0.046)
	TXRV	Diversity	Original pixel	0.909 (0.025)	0.984 (0.005)
			TXRV	0.696 (0.051)	0.932 (0.018)
		Uncertainty	Original pixel	0.888 (0.036)	0.972 (0.013)
			TXRV	0.666 (0.050)	0.926 (0.020)
		Hybrid	Original pixel	0.940 (0.024)	0.989 (0.006)
			TXRV	0.678 (0.050)	0.929 (0.019)
	REMEDIS	Diversity	Original pixel	0.745 (0.040)	0.949 (0.013)
			REMEDIS	0.705 (0.064)	0.926 (0.019)
		Uncertainty	Original pixel	0.790 (0.041)	0.958 (0.013)
			REMEDIS	0.796 (0.041)	0.957 (0.016)
		Hybrid	Original pixel	0.963 (0.015)	0.994 (0.003)
			REMEDIS	0.636 (0.068)	0.899 (0.026)
	DenseNet	Diversity	Original pixel	0.860 (0.034)	0.970 (0.011)
			DenseNet	0.567 (0.058)	0.879 (0.029)
		Uncertainty	Original pixel	0.832 (0.047)	0.942 (0.024)
			DenseNet	0.548 (0.057)	0.891 (0.025)
		Hybrid	Original pixel	0.846 (0.043)	0.970 (0.010)
			DenseNet	0.604 (0.058)	0.884 (0.030)
	ResNet	Diversity	Original pixel	0.897 (0.029)	0.980 (0.008)
			ResNet	0.774 (0.039)	0.954 (0.012)
		Uncertainty	Original pixel	0.888 (0.043)	0.974 (0.012)
			ResNet	0.738 (0.048)	0.939 (0.019)
		Hybrid	Original pixel	0.908 (0.040)	0.978 (0.011)
			ResNet	0.733 (0.046)	0.938 (0.020)
20	/	Random	Original pixel	0.874 (0.129)	0.972 (0.039)
			TXRV	0.775 (0.142)	0.942 (0.044)
			REMEDIS	0.792 (0.146)	0.947 (0.047)
			DenseNet	0.740 (0.106)	0.931 (0.036)
			ResNet	0.750 (0.111)	0.939 (0.039)
	TXRV	Diversity	Original pixel	0.933 (0.021)	0.989 (0.004)
			TXRV	0.948 (0.018)	0.991 (0.003)
		Uncertainty	Original pixel	0.877 (0.034)	0.972 (0.011)
			TXRV	0.819 (0.046)	0.948 (0.020)
		Hybrid	Original pixel	0.944 (0.018)	0.991 (0.003)
			TXRV	0.952 (0.018)	0.992 (0.003)
	REMEDIS	Diversity	Original pixel	0.859 (0.048)	0.968 (0.014)
			REMEDIS	0.880 (0.028)	0.980 (0.006)
		Uncertainty	Original pixel	0.948 (0.024)	0.990 (0.005)
			REMEDIS	0.733 (0.044)	0.931 (0.022)
		Hybrid	Original pixel	0.878 (0.040)	0.973 (0.012)
			REMEDIS	0.954 (0.013)	0.993 (0.002)
	DenseNet	Diversity	Original pixel	0.948 (0.024)	0.989 (0.006)
			DenseNet	0.794 (0.044)	0.960 (0.012)
		Uncertainty	Original pixel	0.888 (0.048)	0.970 (0.016)
			DenseNet	0.824 (0.032)	0.970 (0.008)
		Hybrid	Original pixel	0.970 (0.012)	0.995 (0.002)
			DenseNet	0.885 (0.029)	0.973 (0.011)
	ResNet	Diversity	Original pixel	0.946 (0.023)	0.990 (0.005)
			ResNet	0.900 (0.039)	0.977 (0.011)
		Uncertainty	Original pixel	0.872 (0.044)	0.962 (0.018)
			ResNet	0.861 (0.030)	0.975 (0.007)
		Hybrid	Original pixel	0.930 (0.026)	0.987 (0.006)
			ResNet	0.879 (0.030)	0.977 (0.008)
30	/	Random	Original pixel	0.899 (0.109)	0.977 (0.032)
			TXRV	0.844 (0.109)	0.958 (0.033)
			REMEDIS	0.825 (0.125)	0.956 (0.038)
			DenseNet	0.814 (0.099)	0.955 (0.030)
			ResNet	0.792 (0.103)	0.950 (0.033)
	TXRV	Diversity	Original pixel	0.945 (0.019)	0.991 (0.004)
			TXRV	0.954 (0.014)	0.993 (0.002)
		Uncertainty	Original pixel	0.895 (0.033)	0.977 (0.009)
			TXRV	0.896 (0.039)	0.960 (0.023)
		Hybrid	Original pixel	0.896 (0.034)	0.981 (0.007)
			TXRV	0.937 (0.025)	0.987 (0.005)
	REMEDIS	Diversity	Original pixel	0.832 (0.057)	0.958 (0.017)
			REMEDIS	0.761 (0.054)	0.934 (0.022)
		Uncertainty	Original pixel	0.960 (0.020)	0.992 (0.004)
			REMEDIS	0.848 (0.043)	0.966 (0.012)
		Hybrid	Original pixel	0.909 (0.026)	0.984 (0.006)
			REMEDIS	0.919 (0.026)	0.985 (0.007)
	DenseNet	Diversity	Original pixel	0.963 (0.014)	0.994 (0.002)
			DenseNet	0.847 (0.036)	0.971 (0.009)
		Uncertainty	Original pixel	0.891 (0.048)	0.972 (0.013)
			DenseNet	0.777 (0.050)	0.944 (0.020)
		Hybrid	Original pixel	0.973 (0.009)	0.996 (0.002)
			DenseNet	0.900 (0.023)	0.983 (0.005)
	ResNet	Diversity	Original pixel	0.910 (0.038)	0.978 (0.012)
			ResNet	0.817 (0.049)	0.963 (0.013)
		Uncertainty	Original pixel	0.925 (0.028)	0.985 (0.006)
			ResNet	0.688 (0.042)	0.942 (0.013)
		Hybrid	Original pixel	0.943 (0.019)	0.990 (0.004)
			ResNet	0.852 (0.038)	0.970 (0.010)
40	/	Random	Original pixel	0.931 (0.074)	0.986 (0.020)
			TXRV	0.880 (0.093)	0.967 (0.028)
			REMEDIS	0.853 (0.101)	0.963 (0.036)
			DenseNet	0.860 (0.074)	0.965 (0.025)
			ResNet	0.825 (0.096)	0.958 (0.030)
	TXRV	Diversity	Original pixel	0.951 (0.018)	0.992 (0.004)
			TXRV	0.947 (0.024)	0.991 (0.004)
		Uncertainty	Original pixel	0.918 (0.038)	0.978 (0.012)
			TXRV	0.868 (0.043)	0.951 (0.025)
		Hybrid	Original pixel	0.974 (0.009)	0.996 (0.002)
			TXRV	0.973 (0.010)	0.996 (0.002)
	REMEDIS	Diversity	Original pixel	0.956 (0.021)	0.991 (0.005)
			REMEDIS	0.835 (0.028)	0.974 (0.007)
		Uncertainty	Original pixel	0.953 (0.024)	0.990 (0.005)
			REMEDIS	0.821 (0.049)	0.953 (0.016)
		Hybrid	Original pixel	0.944 (0.030)	0.989 (0.007)
			REMEDIS	0.927 (0.029)	0.983 (0.009)
	DenseNet	Diversity	Original pixel	0.945 (0.021)	0.990 (0.004)
			DenseNet	0.879 (0.035)	0.964 (0.020)
		Uncertainty	Original pixel	0.882 (0.047)	0.970 (0.013)
			DenseNet	0.780 (0.031)	0.958 (0.013)
		Hybrid	Original pixel	0.971 (0.013)	0.995 (0.003)
			DenseNet	0.912 (0.020)	0.985 (0.004)
	ResNet	Diversity	Original pixel	0.914 (0.040)	0.981 (0.010)
			ResNet	0.815 (0.050)	0.957 (0.015)
		Uncertainty	Original pixel	0.936 (0.030)	0.987 (0.007)
			ResNet	0.723 (0.036)	0.949 (0.012)
		Hybrid	Original pixel	0.962 (0.017)	0.993 (0.004)
			ResNet	0.861 (0.036)	0.972 (0.010)
50	/	Random	Original pixel	0.947 (0.042)	0.990 (0.010)
			TXRV	0.890 (0.085)	0.970 (0.027)
			REMEDIS	0.880 (0.075)	0.972 (0.029)
			DenseNet	0.880 (0.067)	0.972 (0.021)
			ResNet	0.843 (0.079)	0.963 (0.025)
	TXRV	Diversity	Original pixel	0.985 (0.007)	0.998 (0.001)
			TXRV	0.932 (0.024)	0.987 (0.005)
		Uncertainty	Original pixel	0.931 (0.025)	0.986 (0.006)
			TXRV	0.901 (0.036)	0.971 (0.017)
		Hybrid	Original pixel	0.990 (0.006)	0.998 (0.001)
			TXRV	0.963 (0.017)	0.993 (0.004)
	REMEDIS	Diversity	Original pixel	0.960 (0.014)	0.993 (0.003)
			REMEDIS	0.947 (0.017)	0.991 (0.004)
		Uncertainty	Original pixel	0.948 (0.027)	0.989 (0.006)
			REMEDIS	0.754 (0.029)	0.956 (0.011)
		Hybrid	Original pixel	0.972 (0.011)	0.996 (0.002)
			REMEDIS	0.915 (0.016)	0.988 (0.003)
	DenseNet	Diversity	Original pixel	0.970 (0.013)	0.995 (0.003)
			DenseNet	0.940 (0.021)	0.989 (0.005)
		Uncertainty	Original pixel	0.959 (0.024)	0.992 (0.005)
			DenseNet	0.767 (0.028)	0.961 (0.008)
		Hybrid	Original pixel	0.973 (0.011)	0.996 (0.002)
			DenseNet	0.875 (0.038)	0.961 (0.020)
	ResNet	Diversity	Original pixel	0.946 (0.026)	0.988 (0.007)
			ResNet	0.815 (0.047)	0.955 (0.016)
		Uncertainty	Original pixel	0.928 (0.026)	0.987 (0.006)
			ResNet	0.805 (0.039)	0.966 (0.009)
		Hybrid	Original pixel	0.978 (0.009)	0.997 (0.002)
			ResNet	0.872 (0.036)	0.974 (0.009)

**Table A3:** Subsequent learning performance of random sampling, diversity, uncertainty, and hybrid methods on the Guangzhou data set.

Overall budget	Foundation model	Initialization method	Model input	AUROC	AUPRC
All samples	/	/	Original pixel	0.998 (0.001)	0.999 (0.000)
			TXRV	0.938 (0.007)	0.977 (0.003)
			REMEDIS	0.993 (0.003)	0.997 (0.002)
			DenseNet	0.848 (0.012)	0.939 (0.007)
			ResNet	0.990 (0.003)	0.996 (0.001)
10 + 10	/	Random	Original pixel	0.938 (0.032)	0.974 (0.014)
			TXRV	0.689 (0.090)	0.848 (0.052)
			REMEDIS	0.882 (0.083)	0.938 (0.057)
			DenseNet	0.853 (0.076)	0.927 (0.041)
			ResNet	0.869 (0.052)	0.938 (0.032)
	TXRV	Diversity	Original pixel	0.974 (0.004)	0.989 (0.003)
			TXRV	0.476 (0.022)	0.704 (0.019)
		Uncertainty	Original pixel	0.913 (0.008)	0.968 (0.004)
			TXRV	0.460 (0.016)	0.728 (0.018)
		Hybrid	Original pixel	0.908 (0.008)	0.965 (0.005)
			TXRV	0.602 (0.017)	0.806 (0.014)
	REMEDIS	Diversity	Original pixel	0.936 (0.006)	0.977 (0.003)
			REMEDIS	0.927 (0.008)	0.968 (0.005)
		Uncertainty	Original pixel	0.893 (0.011)	0.943 (0.009)
			REMEDIS	0.884 (0.012)	0.926 (0.012)
		Hybrid	Original pixel	0.970 (0.005)	0.988 (0.002)
			REMEDIS	0.872 (0.015)	0.910 (0.013)
	DenseNet	Diversity	Original pixel	0.956 (0.005)	0.981 (0.004)
			DenseNet	0.867 (0.014)	0.916 (0.012)
		Uncertainty	Original pixel	0.924 (0.008)	0.966 (0.005)
			DenseNet	0.747 (0.015)	0.864 (0.013)
		Hybrid	Original pixel	0.929 (0.008)	0.971 (0.004)
			DenseNet	0.832 (0.013)	0.916 (0.010)
	ResNet	Diversity	Original pixel	0.981 (0.004)	0.993 (0.002)
			ResNet	0.894 (0.010)	0.949 (0.007)
		Uncertainty	Original pixel	0.931 (0.007)	0.975 (0.003)
			ResNet	0.870 (0.012)	0.936 (0.010)
		Hybrid	Original pixel	0.946 (0.006)	0.979 (0.003)
			ResNet	0.895 (0.011)	0.948 (0.008)
10 + 20^a^	/	Random	Original pixel	0.950 (0.031)	0.980 (0.013)
			TXRV	0.745 (0.058)	0.878 (0.035)
			REMEDIS	0.898 (0.073)	0.946 (0.044)
			DenseNet	0.887 (0.049)	0.944 (0.031)
			ResNet	0.896 (0.031)	0.953 (0.020)
	TXRV	Diversity	Original pixel	0.980 (0.004)	0.991 (0.003)
			TXRV	0.777 (0.016)	0.886 (0.013)
		Uncertainty	Original pixel	0.960 (0.005)	0.985 (0.002)
			TXRV	0.497 (0.015)	0.753 (0.017)
		Hybrid	Original pixel	0.964 (0.006)	0.987 (0.003)
			TXRV	0.740 (0.013)	0.883 (0.010)
	REMEDIS	Diversity	Original pixel	0.941 (0.007)	0.976 (0.004)
			REMEDIS	0.896 (0.011)	0.943 (0.009)
		Uncertainty	Original pixel	0.896 (0.012)	0.946 (0.007)
			REMEDIS	0.885 (0.010)	0.931 (0.011)
		Hybrid	Original pixel	0.969 (0.004)	0.989 (0.002)
			REMEDIS	0.915 (0.010)	0.935 (0.012)
	DenseNet	Diversity	Original pixel	0.973 (0.005)	0.990 (0.003)
			DenseNet	0.924 (0.008)	0.969 (0.004)
		Uncertainty	Original pixel	0.932 (0.007)	0.972 (0.004)
			DenseNet	0.756 (0.016)	0.870 (0.014)
		Hybrid	Original pixel	0.942 (0.007)	0.978 (0.003)
			DenseNet	0.928 (0.007)	0.970 (0.004)
	ResNet	Diversity	Original pixel	0.981 (0.004)	0.993 (0.002)
			ResNet	0.938 (0.008)	0.974 (0.004)
		Uncertainty	Original pixel	0.921 (0.008)	0.969 (0.004)
			ResNet	0.891 (0.010)	0.946 (0.009)
		Hybrid	Original pixel	0.963 (0.005)	0.987 (0.002)
			ResNet	0.903 (0.008)	0.957 (0.006)
10 + 30	/	Random	Original pixel	0.958 (0.026)	0.983 (0.011)
			TXRV	0.773 (0.054)	0.894 (0.032)
			REMEDIS	0.909 (0.051)	0.949 (0.038)
			DenseNet	0.910 (0.029)	0.958 (0.018)
			ResNet	0.908 (0.032)	0.959 (0.016)
	TXRV	Diversity	Original pixel	0.961 (0.005)	0.985 (0.002)
			TXRV	0.859 (0.011)	0.938 (0.008)
		Uncertainty	Original pixel	0.935 (0.007)	0.973 (0.004)
			TXRV	0.529 (0.018)	0.779 (0.015)
		Hybrid	Original pixel	0.944 (0.007)	0.979 (0.003)
			TXRV	0.748 (0.012)	0.890 (0.009)
	REMEDIS	Diversity	Original pixel	0.933 (0.008)	0.974 (0.004)
			REMEDIS	0.647 (0.023)	0.736 (0.017)
		Uncertainty	Original pixel	0.932 (0.008)	0.972 (0.004)
			REMEDIS	0.923 (0.008)	0.952 (0.011)
		Hybrid	Original pixel	0.969 (0.005)	0.988 (0.003)
			REMEDIS	0.929 (0.008)	0.959 (0.009)
	DenseNet	Diversity	Original pixel	0.955 (0.005)	0.982 (0.003)
			DenseNet	0.900 (0.009)	0.961 (0.004)
		Uncertainty	Original pixel	0.954 (0.006)	0.980 (0.004)
			DenseNet	0.923 (0.009)	0.949 (0.010)
		Hybrid	Original pixel	0.924 (0.008)	0.971 (0.004)
			DenseNet	0.933 (0.008)	0.973 (0.004)
	ResNet	Diversity	Original pixel	0.977 (0.003)	0.991 (0.002)
			ResNet	0.942 (0.007)	0.977 (0.004)
		Uncertainty	Original pixel	0.949 (0.006)	0.981 (0.003)
			ResNet	0.939 (0.007)	0.976 (0.004)
		Hybrid	Original pixel	0.981 (0.003)	0.993 (0.001)
			ResNet	0.921 (0.007)	0.969 (0.004)
10 + 40	/	Random	Original pixel	0.964 (0.016)	0.986 (0.006)
			TXRV	0.787 (0.043)	0.901 (0.025)
			REMEDIS	0.913 (0.044)	0.950 (0.035)
			DenseNet	0.922 (0.024)	0.964 (0.014)
			ResNet	0.919 (0.027)	0.965 (0.013)
	TXRV	Diversity	Original pixel	0.970 (0.005)	0.987 (0.003)
			TXRV	0.847 (0.011)	0.929 (0.008)
		Uncertainty	Original pixel	0.977 (0.004)	0.990 (0.003)
			TXRV	0.588 (0.018)	0.803 (0.016)
		Hybrid	Original pixel	0.962 (0.006)	0.986 (0.002)
			TXRV	0.739 (0.014)	0.885 (0.010)
	REMEDIS	Diversity	Original pixel	0.948 (0.006)	0.981 (0.003)
			REMEDIS	0.866 (0.013)	0.942 (0.010)
		Uncertainty	Original pixel	0.944 (0.007)	0.978 (0.003)
			REMEDIS	0.928 (0.012)	0.938 (0.014)
		Hybrid	Original pixel	0.967 (0.005)	0.985 (0.004)
			REMEDIS	0.946 (0.009)	0.956 (0.010)
	DenseNet	Diversity	Original pixel	0.980 (0.004)	0.991 (0.002)
			DenseNet	0.932 (0.009)	0.968 (0.006)
		Uncertainty	Original pixel	0.964 (0.005)	0.985 (0.004)
			DenseNet	0.945 (0.006)	0.977 (0.004)
		Hybrid	Original pixel	0.983 (0.003)	0.993 (0.002)
			DenseNet	0.917 (0.007)	0.969 (0.003)
	ResNet	Diversity	Original pixel	0.976 (0.003)	0.991 (0.002)
			ResNet	0.938 (0.009)	0.967 (0.008)
		Uncertainty	Original pixel	0.954 (0.006)	0.983 (0.003)
			ResNet	0.943 (0.006)	0.978 (0.003)
		Hybrid	Original pixel	0.977 (0.004)	0.991 (0.002)
			ResNet	0.922 (0.008)	0.966 (0.006)

^a^ The overall budget consists of both the initialization and subsequent learning components. For instance, a budget of 10 + 20 indicates an initialization budget comprising 10 samples and a subsequent learning budget also comprising 20 samples.

**Table A4:** Subsequent learning performance of random sampling, diversity, uncertainty, and hybrid methods on the Pakistan data set.

Overall budget	Foundation model	Initialization method	Model input	AUROC	AUPRC
All samples	/	/	Original pixel	0.994 (0.003)	0.999 (0.001)
			TXRV	0.991 (0.006)	0.999 (0.001)
			REMEDIS	0.968 (0.016)	0.994 (0.003)
			DenseNet	0.964 (0.023)	0.987 (0.010)
			ResNet	0.993 (0.005)	0.999 (0.001)
10 + 10	/	Random	Original pixel	0.838 (0.125)	0.963 (0.037)
			TXRV	0.795 (0.135)	0.948 (0.038)
			REMEDIS	0.791 (0.176)	0.947 (0.057)
			DenseNet	0.743 (0.103)	0.936 (0.033)
			ResNet	0.757 (0.107)	0.943 (0.031)
	TXRV	Diversity	Original pixel	0.928 (0.018)	0.988 (0.004)
			TXRV	0.574 (0.046)	0.894 (0.032)
		Uncertainty	Original pixel	0.899 (0.030)	0.979 (0.008)
			TXRV	0.866 (0.034)	0.959 (0.021)
		Hybrid	Original pixel	0.945 (0.018)	0.991 (0.003)
			TXRV	0.863 (0.045)	0.950 (0.024)
	REMEDIS	Diversity	Original pixel	0.792 (0.049)	0.958 (0.013)
			REMEDIS	0.872 (0.045)	0.960 (0.019)
		Uncertainty	Original pixel	0.739 (0.046)	0.948 (0.013)
			REMEDIS	0.814 (0.047)	0.939 (0.024)
		Hybrid	Original pixel	0.904 (0.033)	0.981 (0.008)
			REMEDIS	0.965 (0.011)	0.994 (0.002)
	DenseNet	Diversity	Original pixel	0.873 (0.032)	0.975 (0.009)
			DenseNet	0.845 (0.048)	0.950 (0.023)
		Uncertainty	Original pixel	0.910 (0.044)	0.976 (0.012)
			DenseNet	0.746 (0.045)	0.924 (0.027)
		Hybrid	Original pixel	0.847 (0.041)	0.971 (0.010)
			DenseNet	0.830 (0.028)	0.970 (0.007)
	ResNet	Diversity	Original pixel	0.826 (0.054)	0.953 (0.020)
			ResNet	0.848 (0.036)	0.970 (0.008)
		Uncertainty	Original pixel	0.930 (0.028)	0.986 (0.007)
			ResNet	0.796 (0.046)	0.959 (0.011)
		Hybrid	Original pixel	0.940 (0.016)	0.990 (0.004)
			ResNet	0.880 (0.030)	0.979 (0.007)
10 + 20^a^	/	Random	Original pixel	0.889 (0.121)	0.976 (0.033)
			TXRV	0.860 (0.101)	0.966 (0.028)
			REMEDIS	0.840 (0.114)	0.963 (0.033)
			DenseNet	0.787 (0.093)	0.949 (0.030)
			ResNet	0.803 (0.098)	0.955 (0.028)
	TXRV	Diversity	Original pixel	0.956 (0.014)	0.993 (0.003)
			TXRV	0.869 (0.040)	0.972 (0.010)
		Uncertainty	Original pixel	0.904 (0.029)	0.980 (0.008)
			TXRV	0.881 (0.031)	0.972 (0.013)
		Hybrid	Original pixel	0.954 (0.013)	0.992 (0.003)
			TXRV	0.933 (0.031)	0.983 (0.010)
	REMEDIS	Diversity	Original pixel	0.963 (0.012)	0.994 (0.002)
			REMEDIS	0.807 (0.042)	0.954 (0.015)
		Uncertainty	Original pixel	0.790 (0.048)	0.959 (0.012)
			REMEDIS	0.904 (0.037)	0.975 (0.012)
		Hybrid	Original pixel	0.920 (0.033)	0.984 (0.007)
			REMEDIS	0.909 (0.027)	0.981 (0.008)
	DenseNet	Diversity	Original pixel	0.880 (0.029)	0.978 (0.007)
			DenseNet	0.917 (0.020)	0.987 (0.004)
		Uncertainty	Original pixel	0.929 (0.034)	0.984 (0.008)
			DenseNet	0.731 (0.052)	0.912 (0.028)
		Hybrid	Original pixel	0.743 (0.061)	0.941 (0.019)
			DenseNet	0.800 (0.043)	0.951 (0.017)
	ResNet	Diversity	Original pixel	0.976 (0.008)	0.996 (0.001)
			ResNet	0.886 (0.037)	0.962 (0.016)
		Uncertainty	Original pixel	0.936 (0.028)	0.987 (0.007)
			ResNet	0.860 (0.033)	0.975 (0.007)
		Hybrid	Original pixel	0.963 (0.011)	0.994 (0.002)
			ResNet	0.870 (0.036)	0.973 (0.008)
10 + 30	/	Random	Original pixel	0.924 (0.080)	0.985 (0.018)
			TXRV	0.885 (0.065)	0.971 (0.022)
			REMEDIS	0.881 (0.083)	0.974 (0.024)
			DenseNet	0.819 (0.086)	0.955 (0.029)
			ResNet	0.829 (0.085)	0.962 (0.024)
	TXRV	Diversity	Original pixel	0.967 (0.012)	0.995 (0.002)
			TXRV	0.910 (0.034)	0.970 (0.018)
		Uncertainty	Original pixel	0.911 (0.032)	0.981 (0.008)
			TXRV	0.868 (0.037)	0.957 (0.021)
		Hybrid	Original pixel	0.932 (0.019)	0.988 (0.004)
			TXRV	0.947 (0.033)	0.981 (0.014)
	REMEDIS	Diversity	Original pixel	0.967 (0.011)	0.995 (0.002)
			REMEDIS	0.861 (0.037)	0.967 (0.014)
		Uncertainty	Original pixel	0.874 (0.041)	0.973 (0.010)
			REMEDIS	0.728 (0.055)	0.910 (0.030)
		Hybrid	Original pixel	0.918 (0.030)	0.985 (0.007)
			REMEDIS	0.705 (0.058)	0.898 (0.031)
	DenseNet	Diversity	Original pixel	0.899 (0.030)	0.978 (0.009)
			DenseNet	0.885 (0.029)	0.978 (0.008)
		Uncertainty	Original pixel	0.910 (0.038)	0.979 (0.009)
			DenseNet	0.841 (0.031)	0.975 (0.006)
		Hybrid	Original pixel	0.892 (0.032)	0.978 (0.010)
			DenseNet	0.780 (0.044)	0.951 (0.015)
	ResNet	Diversity	Original pixel	0.973 (0.009)	0.996 (0.002)
			ResNet	0.845 (0.039)	0.958 (0.019)
		Uncertainty	Original pixel	0.921 (0.028)	0.985 (0.006)
			ResNet	0.843 (0.042)	0.952 (0.020)
		Hybrid	Original pixel	0.961 (0.011)	0.994 (0.002)
			ResNet	0.879 (0.027)	0.979 (0.006)
10 + 40	/	Random	Original pixel	0.943 (0.042)	0.989 (0.009)
			TXRV	0.906 (0.050)	0.976 (0.019)
			REMEDIS	0.867 (0.100)	0.969 (0.034)
			DenseNet	0.846 (0.068)	0.961 (0.025)
			ResNet	0.854 (0.062)	0.969 (0.018)
	TXRV	Diversity	Original pixel	0.951 (0.015)	0.992 (0.003)
			TXRV	0.923 (0.018)	0.987 (0.004)
		Uncertainty	Original pixel	0.911 (0.025)	0.983 (0.007)
			TXRV	0.836 (0.043)	0.950 (0.020)
		Hybrid	Original pixel	0.919 (0.028)	0.985 (0.006)
			TXRV	0.961 (0.020)	0.993 (0.004)
	REMEDIS	Diversity	Original pixel	0.950 (0.017)	0.992 (0.003)
			REMEDIS	0.920 (0.032)	0.985 (0.006)
		Uncertainty	Original pixel	0.865 (0.034)	0.973 (0.009)
			REMEDIS	0.837 (0.035)	0.966 (0.014)
		Hybrid	Original pixel	0.932 (0.026)	0.987 (0.006)
			REMEDIS	0.959 (0.013)	0.993 (0.002)
	DenseNet	Diversity	Original pixel	0.938 (0.022)	0.990 (0.004)
			DenseNet	0.942 (0.017)	0.990 (0.004)
		Uncertainty	Original pixel	0.935 (0.026)	0.986 (0.007)
			DenseNet	0.849 (0.030)	0.973 (0.007)
		Hybrid	Original pixel	0.970 (0.013)	0.995 (0.002)
			DenseNet	0.851 (0.035)	0.972 (0.008)
	ResNet	Diversity	Original pixel	0.980 (0.009)	0.997 (0.002)
			ResNet	0.934 (0.016)	0.989 (0.003)
		Uncertainty	Original pixel	0.935 (0.023)	0.988 (0.005)
			ResNet	0.885 (0.032)	0.979 (0.007)
		Hybrid	Original pixel	0.982 (0.007)	0.997 (0.001)
			ResNet	0.861 (0.034)	0.974 (0.008)

^a^ The overall budget consists of both the initialization and subsequent learning components. For instance, a budget of 10 + 20 indicates an initialization budget comprising 10 samples and a subsequent learning budget comprising 20 samples.
